# Poly(Lactic Acid)-Based Microparticles for Drug Delivery Applications: An Overview of Recent Advances

**DOI:** 10.3390/pharmaceutics14020359

**Published:** 2022-02-04

**Authors:** Antonios Vlachopoulos, Georgia Karlioti, Evangelia Balla, Vasileios Daniilidis, Theocharis Kalamas, Myrika Stefanidou, Nikolaos D. Bikiaris, Evi Christodoulou, Ioanna Koumentakou, Evangelos Karavas, Dimitrios N. Bikiaris

**Affiliations:** 1 Laboratory of Polymer Chemistry and Technology, Department of Chemistry, Aristotle University of Thessaloniki, GR-541 24 Thessaloniki, Greece; antwnis97@gmail.com (A.V.); geocar1997@gmail.com (G.K.); euagelia226@gmail.com (E.B.); basdanil17@gmail.com (V.D.); xkalamas@gmail.com (T.K.); myroforastefanidou@gmail.com (M.S.); nbikiaris@gmail.com (N.D.B.); evicius@gmail.com (E.C.); iwanna.koumentakou@gmail.com (I.K.); 2 Pharmathen S.A., Pharmaceutical Industry, Dervenakion Str. 6, Pallini Attikis, GR-153 51 Attiki, Greece

**Keywords:** poly(lactic acid), microparticles, drug delivery, preparation techniques, drug release mechanisms, copolymers

## Abstract

The sustained release of pharmaceutical substances remains the most convenient way of drug delivery. Hence, a great variety of reports can be traced in the open literature associated with drug delivery systems (DDS). Specifically, the use of microparticle systems has received special attention during the past two decades. Polymeric microparticles (MPs) are acknowledged as very prevalent carriers toward an enhanced bio-distribution and bioavailability of both hydrophilic and lipophilic drug substances. Poly(lactic acid) (PLA), poly(lactic-co-glycolic acid) (PLGA), and their copolymers are among the most frequently used biodegradable polymers for encapsulated drugs. This review describes the current state-of-the-art research in the study of poly(lactic acid)/poly(lactic-co-glycolic acid) microparticles and PLA-copolymers with other aliphatic acids as drug delivery devices for increasing the efficiency of drug delivery, enhancing the release profile, and drug targeting of active pharmaceutical ingredients (API). Potential advances in generics and the constant discovery of therapeutic peptides will hopefully promote the success of microsphere technology.

## 1. Introduction

Advances in pharmaceutical and other associated fields demand site-specific delivery of drugs, vaccines, genes, and many other biomolecules. Furthermore, for the effective treatment of a disease, the stability and safety issues related to these agents may be challenging during the development and storage of advanced marketed products [1,2]. New drug delivery formulations and their applications have been studied for a long time, with the current focus being on microparticle systems and their benefits. The definition of the term “microparticle” is a spherical particle with a size from 1 μm to 2 mm containing a core substance enclosed by one or more membranes or shells. Microparticles may be further classified as microspheres and microcapsules based on their internal structure. Microspheres are generally formed by a homogeneous matrix in which it is not possible to separate a core and a membrane, while the API is dispersed in the polymer matrix either as small clusters or molecularly. Microcapsules are formulations constituted by a central liquid, solid, or semisolid core containing the API, alone, or in combination with excipients, surrounded by a membrane or a continuous polymer coating [3]. Stretching the definition of a microcapsule, we can include not only membrane enclosed particles or droplets but also solid matrix dispersions without external membranes or wall structures. In order to select the appropriate material for the capsule wall or the matrix, characteristics such as film formation, hydrophilicity and hydrophobicity, release profile, and degradation curve must be considered [4].

As a drug delivery system, MPs overcome the disadvantages of the traditional dosage forms and offer many advantages, such as the use of different administration routes or the opportunity of encapsulating various molecules, reduced toxicity, effective and accurate control over long periods, as well as the protection of the encapsulated agent against oxidation and the ease of administration to people that are unable to provide for themselves, such as children and people with special needs. MPs can also be used for the controlled release of drugs that can be adapted by the choice of polymer and its chemical and molecular features, such as molecular weight (Mw), monomer composition, crystallinity, glass transition temperature (Tg), and inherent viscosity [1,5]. These systems consist of a biocompatible polymeric material that allows for control over drug release and protection of the drug cargo [6]. Furthermore, besides drugs, microparticles are ideal candidates for peptide and protein administration. The high molecular weight and polar nature of peptides and proteins results in low membrane permeability, while the structural sensitivity of proteins constitutes their incompatible with the gastrointestinal tract. Because of this, peptides and proteins are usually delivered as injectable formulations. While humanized antibodies may be long circulating in the bloodstream, peptide therapeutics can be cleared from the organism in a matter of minutes either due to enzymatic degradation or renal clearance. Thus, an effective delivery and prolonged release of biologics demands the encapsulation of these agents into therapeutic MPs [7].

A MP depot system is suitable when the following requirements are being achieved: (1) the stability of the encapsulated active ingredient is maintained; (2) an optimal drug loading is obtained; (3) a high encapsulation efficiency and yield is accomplished; (4) desired drug release profiles with low initial release are attained; (5) particles of free-flowing and good syringeability are produced; (6) and a simple, scalable, and reproducible process is established. Some features, such as morphological characteristics, particle size, and polydispersity index (PDI), are essential to ensure stability, encapsulation, and a sufficient release of the drug, and they are directly related to the preparation technique of the MPs. Thus, preparation techniques are selected based on the drug in question and according to the specific application of the microparticle formulation [5]. A brief discussion on the most common techniques is provided below. Recent advancements in MP formulations suggest the use of biodegradable polymers for the effective delivery of proteins, peptides, and other biomolecules over inorganic materials due to potential toxicity on the human body and adverse effects on the environment [3]. Furthermore, polymers enable modification in (a) physicochemical properties (e.g., hydrophobicity, zeta potential), (b) drug release profiles (e.g., delayed, prolonged, triggered), and (c) biological properties (e.g., bioadhesion, improved cellular uptake) of the MPs [8]. Biobased and biodegradable polymers, such as poly(lactic acid) PLA, poly(glycolic acid) (PGA), poly(lactic-co-glycolic acid) (PLGA), poly(hydroxy alkanoates) (PHA), poly(ε-caprolactone) (PCL), and their copolymers, are considered ideal materials for microencapsulated drug delivery systems. They are easily absorbable and can be naturally decomposed through enzymatic or nonenzymatic processes that result in biocompatible and toxicologically safe for humans byproducts, which are then eliminated by the normal metabolic pathways [3]. In addition, they exhibit interesting physical properties and various erosion times, while the fact that they have been approved by the Food and Drug Administration (FDA) has generally facilitated their use as drug delivery systems and biomaterials [9]. Polymeric microparticles are also starting to be used in the field of vaccination. MPs can overcome the obstacles derived from the use of needles and are more patient-friendly. A promising painless and controlled system for drug delivery is microneedle patches. Those usually contain micro-particles able to penetrate skin [10].

Among the above-mentioned biopolymers, the advantages offered by PLA have led researchers to broadly implement its usage in many applications, including drug delivery systems. The environmental friendliness, the ease of production, the recyclability, compostability, and biocompatibility and the absence of carcinogenic effects are a few of its benefits. In addition, PLA can be obtained from renewable resources such as wheat, corn, and rice; thus, its production requires 25–55% less energy than conventional petrol deriving polymers. Lastly, PLA’s degradation products are also non-toxic to human applications and the environment [11].

Recently, old challenges have been addressed from new perspectives, driven by advances in related disciplines aiming to take microencapsulation technology one step forward [12]. This review article aims to present the latest microencapsulated formulations based on biodegradable and biocompatible polymers, such as PLA, PLGA, and their copolymers, on a research level.

## 2. MP Preparation Techniques at a Glance

Throughout the years, a diverse number of techniques have been developed and can be employed for the preparation of microparticles for drug delivery applications, leading to a great variety of morphologies, structures, and size ranges. They are based on either physicochemical (i.e., emulsion solvent evaporation methods, electrospraying), chemical (i.e., polymerization), or mechanical (i.e., spray drying, microfluidics, supercritical fluid) processes.

**Figure 1:**
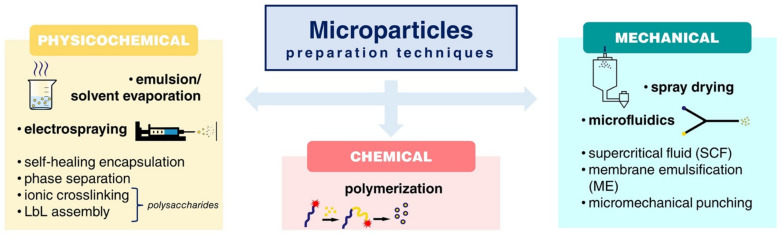
A schematic overview of different techniques for MP preparation.

The most frequently used approach for nano-/microparticles manufacturing is emulsification–solvent evaporation (including double or multiple emulsions). It is a simple, low-cost, fast, and reproducible technique that allows for adjustable particle size by altering the viscosity of organic/aqueous phases, the homogenization speed, and the concentration of the emulsifier. The general principle is the emulsification of a polymer solution (dissolved in an organic solvent, typically chloroform or dichloromethane) in an aqueous continuous phase (supplied with an emulsifier, e.g., poly(vinyl alcohol)) by mechanical agitation until the solvent partitions into the aqueous phase and is removed by evaporation. The microspheres are then recovered by centrifugation and/or filtration, and lyophilized. The single oil-in-water (o/w) emulsion technique (Figure 1) is generally applied for the encapsulation of hydrophobic or poorly water-soluble active ingredients. In the case of hydrophilic agents, which suffer from low encapsulation efficiency because of rapid drug partitioning into the external aqueous phase when using single emulsions, double or multiple emulsions (most commonly a water-in-oil-in-water, w/o/w, emulsion) are adopted [8]. However, the large amounts of organic solvents required, the narrow and uniform size distributions of the particles obtained, and the difficulty of scaling up are the main drawbacks that limit the use of this process at the industrial level [13].

**Figure 2:**
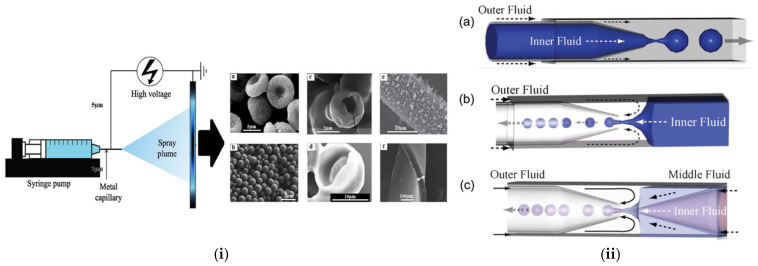
(**i**) Different structures prepared by the electrospraying method: (**a**) hollow sphere, (**b**) spherical particle with smooth surface, (**c**) hollow particles, (**d**) electrospray droplets (**f**) electrically conducting substrates and (**e**) enteric coated particles. (**ii**) Schematic demonstration of microfluidic devices for fabricating monodisperse microparticles: (**a**) co-flow capillary device, (**b**) flow-focusing capillary device, and (**c**) a double emulsion capillary microfluidic device that combines co-flow and flow focusing. Adapted with permission from ref. [22,23]. 2021, Elsevier.

Spray drying and electrospray represent two recent and appealing strategies for microencapsulation of active compounds in the pharmaceutical, food, and cosmetic fields. The spray drying mechanism is based on the atomizing and subsequent drying of a feed (i.e., a liquid, S/O or W/O, dispersion of particles solution) by spraying it into a hot drying medium (air, inert gas, such as nitrogen). The process is generally divided into three steps: (i) atomization of the feed into small droplets via an atomizer, (ii) drying of the droplets upon contact with the drying gas and particle formation, and (iii) separation of the dry particles from the drying medium [14]. Lately, the technique has been successfully employed for the preparation of inhalable formulations suitable for pulmonary drug delivery [15,16]. However, adhering particles to the inner wall of the spray-dryer is the major drawback [13]. Electrospray (ES) (Figure 2i) has also been recently introduced as a low-cost and effective alternative approach for MP production, offering greater control over particle size, higher drug encapsulation efficiency, less residue generation, and the need for smaller amounts of solvents [17]. It is an electrohydrodynamic process in which monodisperse droplets are formed by leading a liquid of sufficient electrical conductivity through a capillary channel or nozzle to a high potential. The final MP size can be tuned by many variable parameters, such as the electrostatic field strength, the needle size, the solution flow rate, the concentration etc. [18].

In the same sense, microfluidic technologies are currently a powerful tool to generate microparticles with high monodispersity, precisely tunable structures, and excellent encapsulation efficiency [19]. The technique enables precise manipulation of micro-flows in the channels of a micronized chip to produce uniform picoliter emulsion droplets [20]. Microfluidic chips are either lab-made or commercial products made of glass, polymers, or polydimethylsiloxane (PDMS). They can be classified into co-flow capillary devices, flow-focusing capillary devices, and the combination of these two principles (Figure 2ii). Despite the limited production scale of a single microfluidic device, scaling up of this technology is feasible by simultaneously operating multiple microfluidic devices in parallel [21].

Recently, the supercritical fluid (SCF) method has also been extensively applied to prepare nano/microparticles. Among the various SCFs used, supercritical carbon dioxide (scCO_2_) has received special attention as an alternative green candidate for the replacement of conventional organic solvents and the implementation of mild, environmentally friendly process conditions. The scCO_2_, with its relatively low critical temperature (31.1 °C) and critical pressure (73.8 bar), presents the unique ability to dissolve certain polymers [24]. During the SCF methods, the particles are formed by the rapid decompression occurring due to the depressurization of a polymer solution (priory dissolved in scCO_2_) through a nozzle into a lower pressure environment. Again, the pressure, nozzle diameter, solution concentration, and temperature are critical process parameters that can affect the final particle properties. There is currently a broad variety of techniques involving supercritical fluids (Table 1), some of them being rapid expansion of supercritical solutions (RESS), gas antisolvent process (GAS), supercritical antisolvent process (SAS), and solution enhanced dispersion by supercritical fluids (SEDS) [25]. The selection of the most appropriate processing technique depends greatly on the interaction of the supercritical CO_2_ with the active ingredient, the polymeric coating material of interest, and a suitable solvent [26].

Recent advancements in microparticle manufacturing technology include procedures such as membrane emulsification (ME) [28], active self-healing encapsulation (ASE) [10], and micromechanical punching (MMP) for the fabrication of non-spherical microparticles [29]. Finally, ionotropic cross-linking [29,30] and LbL-assembly [31,32] are commonly used strategies in polysaccharide-based micro-sized drug delivery systems.

## 3. Overview of Release Mechanisms

**Figure 3:**
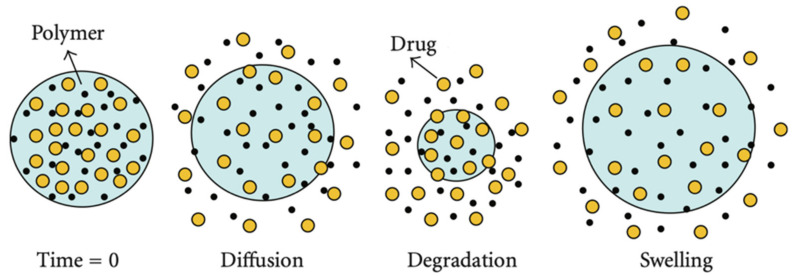
Drug release mechanisms of PLA microspheres [39].

The term “release mechanism” can be described as the way in which drug molecules are released or transported and as a description of the process or event that controls the release rate [33]. Researchers, in most cases, use the term release mechanism when referring to the process that determines the release rate of the drug. Consequently, describing the process of monitoring the release rate is more enlightening than describing the method of drug release when it comes to how drug release can be modified [34]. Factors such as polymer composition (glycolic/lactic acid ratio), pH, hydrophilicity in the backbone, water absorption, glass transition temperature (Tg), and average molecular weight can modulate the degradation rate and erosion of the particles [35]. From the above, it is clear that data on the physicochemical processes that influence the release rate and knowledge of the release mechanisms are of great significance prior to developing controlled-release DDSs [36,37]. There are only three possible ways of drug release from a PLA-based DDS: (i) transport through the polymer, (ii) transport through water-filled pores, and (iii) due to dissolution of the encapsulating polymer (which does not require drug transport). Most commonly, the encapsulated drug is a large molecule of protein or peptide, too large and too hydrophilic to be transported through the polymer phase. For this reason, transport through water-filled pores is the most common way of release observed in PLA and PLGA microparticle systems. Transport through the polymer phase may happen when the drug is hydrophobic and small [34]. In most cases, more than one mechanism takes place in microparticle drug delivery systems. The three basic ways of drug release lead to three different mechanisms in drug release from polymer microparticle systems: (i) release through diffusion from the surface of particles, (ii) release through erosion of particles due to polymer degradation, and (iii) release through the swollen polymer matrix, as illustrated in Figure 3 [35,38].

### 3.1. Diffusion

Diffusion takes place when drug molecules are dissolved in fluids of the human body around or within the particles and migrate away from the particles [39]. The release rate is frequently considered to be diffusion controlled initially and degradation/erosion controlled during the last stage of the release period [40]. Almost always in a drug delivery system, diffusional mass transport is involved. In various cases, the ascendent step is drug diffusion, while in others it “only” plays a major role, e.g., in combination with polymer swelling or polymer degradation/matrix erosion. In specific cases, it even constitutes only a negligible role. Fick’s laws of diffusion can be used to quantify the diffused mass during the release (Equation (1)). Fick’s first law of diffusion:

(1) $$F=-D\frac{\partial c}{\partial x}$$

where F is the rate of transfer per unit area of section (flux); c is the concentration of the diffusing species and denotes the diffusion coefficient (also called diffusivity). Fick’s second of diffusion can be derived from the first one and mass balance considerations (Equation (2)):

(2) $$\frac{\partial c}{\partial x}=D\left(\frac{\partial^{2}c}{\partial x^{2}}+\frac{\partial^{2}c}{\partial y^{2}}+\frac{\partial^{2}c}{\partial z^{2}}\right)$$

where c denotes the concentration of the diffusing species; t is time, D stands for the diffusion coefficient; and x, y, and z are the three spatial (Cartesian) coordinates [41,42]. Porous microparticles from PLLA/PDLA 7/3 ratio prepared by Yu et al. manifested rapid initial release of rifampicin compared to microspheres without pores, since the cracks on the surface can considerably boost the diffusive escape of the drug and endorse the hydrolysis of amorphous PLA. In addition, it was observed that a closed surface significantly delays the diffusion of rifampicin from particles, while the relatively low specific surface area and high molecular weight of PLLA cause hydrolysis speed to be low, thus delaying the release of rifampicin as well. In conclusion, the drug release behavior of PLA microspheres can be adjusted by simply modifying the ratio of PLLA to PDLA. Therefore, the combination of various types of PLLA/PDLA microspheres can regulate the drug release rate based on the actual demands [43].

### 3.2. Erosion

Erosion, i.e., mass loss of the polymer, is initiated when the dissolved polymer degradation products are able to diffuse into the release area [38]. PLGA is usually subjected to bulk erosion, in contrast to surface erosion, as PLGA is relatively swiftly hydrated. Dissolution of polymer degradation products and erosion lead to pore formation. Gradually, the size of the formed pores begins to increase, as water causes hydrolysis, and the produced acids catalyze degradation and cause polymer dissolution inside the pores, leading to subsequent erosion. Small pores consequently grow, and eventually join neighboring pores to form fewer, larger pores [38,44]. Degradation occurs when the polymer chains hydrolyze into lower molecular weight chains, successfully releasing drug molecules that are confined by the polymer chains [39]. Hydrolysis, i.e., the scission of ester bonds and successive decrease in Mw, starts immediately when water or aquatic medium penetrates into the drug delivery devices [45,46]. In an in vivo environment, the polyester backbone structures of PLA and PLGA experience hydrolysis and generate biocompatible components (glycolic acid and lactic acid) that are eradicated from the human body through the citric acid cycle. Normal physiological functions are not affected by the degradation products [35]. This autocatalytic phenomenon is known to induce heterogeneous degradation inside PLGA matrice i.e., quicker degradation at the center of the PLGA matrix than at the shell, since acid oligomers are entrapped in the center of microparticles [47,48]. For the biodegradable polymer matrix, release is usually controlled by the hydrolytic cleavage of polymer chains that result in matrix erosion, even though diffusion might still be dominant when the erosion is slow [49].

### 3.3. Swelling

**Figure 4:**
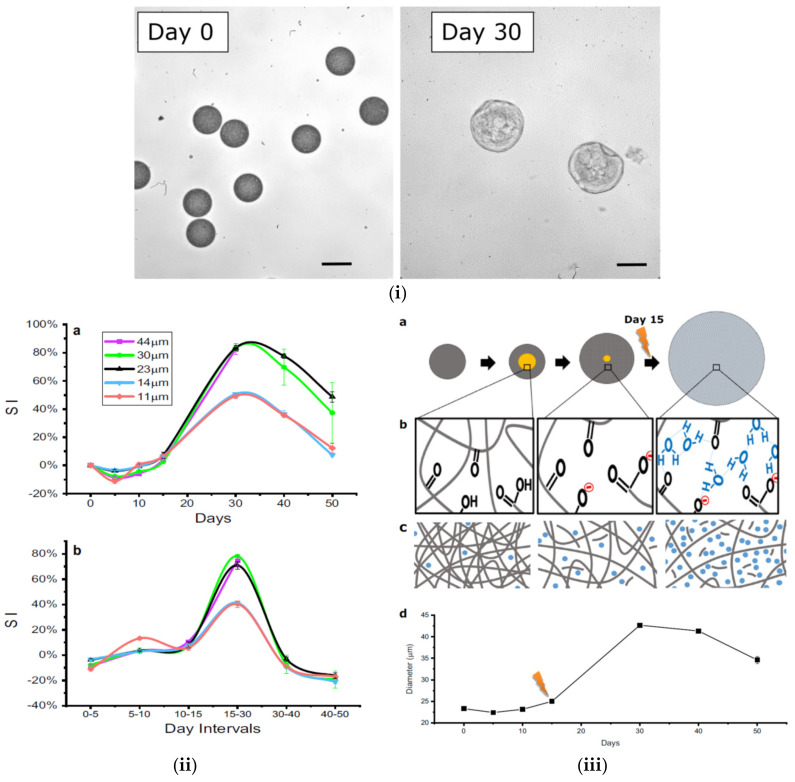
(**i**) Microparticle (44 μm) swelling changes over time, on day 0 and swollen microparticles (79 μm) on day 30. (**ii**) Swelling kinetics: (**a**) Complete swelling index (SI) changes from day 0. (**b**) Swelling changes between each incubation time interval. (**iii**) Interplay of expansive intermolecular forces: (**a**) Schematic of microparticle swelling over time. (**b**) Insets represent the hydrolysis, degradation, hydration shell, and swelling that occurs over time in terms of active chemistry. (**c**) General view of water ingress over time and subsequent hydrolysis of the polymer, (**d**) Swelling profile timeline as it correlates with the schematic (taken from 23 μm population data) [50].

In some cases, apart from diffusion and matrix erosion of microspheres, the release mechanism is more complicated, and in a recent study, the swelling behavior of PLGA microparticles was further investigated [50]. Swelling-controlled release systems were originally dry. PLGA systems with agile polymer chains, when located inside the body, tend to absorb a significant quantity of water, and swell to grow inside pressure and porosity, allowing drug molecules to diffuse from the swollen network. As the volume of water inside increases over time, any noteworthy uprise in pressure will probably be compensated for by swelling and rearrangement of the polymer chains. The release of active drug molecules can also be varied over a specific period of time based on external and internal factors [34,39]. In the case of initially porous system surfaces, release of the drug molecules through these pores might be rapid at the beginning, leading to a “burst effect”. However, polymer swelling can close these pores and subsequently slow down the process of drug release. Furthermore, swelling has also been acknowledged as the main reason for the onset of the final rapid drug release phase from various systems of PLGA-based microparticles. Usually, the latter exhibit tri-phasic drug release patterns; an initial rapid release phase (“burst release”), followed by a phase with an approximately stable drug release rate (sometimes even close to zero), and a final, again rapid drug release phase (leading to complete drug exhaust) has been observed in studies. Scrutinizing the swelling and drug release behavior of “single microparticles” indicated that the onset of substantial microparticle swelling (after a certain lag time) concurred with the onset of the final rapid drug release phase [42,51]. Microparticles were incubated at 37 °C in phosphate buffered saline (PBS) solution (pH 7.4), and their swelling was observed for up to 50 days. It appeared that microparticles started to decrease in size after 5 days of incubation and gained their original size after 10 days of incubation due to water absorption. The swelling index is lower in microparticles with diameter size < 15 μm (49–51%) and much higher for microparticles with diameter sizes > 15 μm (82–83%). In all studied microparticles, their diameter at days between 15 and 30 started to increase, reaching their highest size by day 30, and after that time, a reduction was observed. (Figure 4i). However, since PLGA oligomers with molecular weights around 1100 are water soluble [48], they can slowly escape out of the particles through a diffusion-controlled mechanism, and thus a dissipation of the acidic core was eventually reported after 15 days (Figure 4(iib)). Microchannels may form, and thus water can enter inside microparticles (Figure 4(iiib)). With swelling starting after the 15th day and lasting up to 30 days, when surface erosion takes place progressively.

## 4. Recent Advances in Microparticle-Based Delivery Systems

### 4.1. PLA Microparticles

PLA offers several advantages, such as biodegradability and biocompatibility, which constitute an ideal vehicle for parenteral controlled drug delivery systems. Furthermore, PLA microparticles can control drug release rates for several time periods, lasting from a few days to several weeks up to a year depending on the molecular weight of PLA, MP size, drug loading, solubility, and diffusion ability [52]. PLA microparticles can be easily prepared, mainly by emulsion solvent evaporation techniques, and after solvent removal microspheres are hardening encapsulating hydrophilic or hydrophobic drugs. Drug release is mainly controlled by diffused mechanisms from the insoluble matrix [53]. Several drugs have been encapsulated in PLA MPs in recent years, aiming at their storage stability, enhanced bioavailability, and sustained or prolonged release profiles [54].

Mildronate is a cardioprotective drug with a highly hygroscopic nature and excellent bioavailability. Its hygroscopic character means that the pharmaceutical product requires specific package requirements and storage conditions. To overcome these inconveniences, Loca et al. studied the microencapsulation of mildronate in PLA matrices through a double emulsion application [55]. The PLA drug-loaded microcapsules were comprised of a fairly homogenous mixture of both polymer and drug. PLA was found to act as a watertight coating membrane, and long-term, it decreased the hygroscopicity of mildronate by more than two times, whereas the physical state of the drug and its in vivo release behavior were not substantially affected. In another case, the conjugation of drugs onto the distal -OH end groups of the PLA backbone was reported to prepare pharmacologically active polymeric systems that impart enhanced solubility and stability of the conjugates and provide an opportunity for combination drug delivery [56].

PLA injectable microparticle formulations were found to prolong drug release behavior compared to their PLGA counterparts in a study where bupivacaine drug has been encapsulated to be used as a local anesthetic [57]. In addition, it was found that drug release is directly dependent on particle size and drug feed ratios. Microparticles with higher drug loading and sizes have much higher prolonged periods.

In general, the molecular weight of PLA is a crucial factor during the encapsulation process of active ingredients. In that sense, Chaiyasat et al. worked on the encapsulation of Vitamin E on poly(l-lactic acid) (PLLA) microspheres prepared by the oil-in-water emulsion/solvent evaporation technique, while changing the PLLA/Vitamin E weight ratio and also by using different molecular weights of PLLA [58]. It was found that the optimum ratio for the formation of microparticles was 25:1 PLLA/Vitamin E, whereas low molecular weight PLLA exhibited a poorer carrier capacity compared to higher molecular weight PLLA that could better envelop the vitamin in its interior.

Addressing the issue of drug solubility in a recent study, paliperidone, an antipsychotic drug used in patients suffering from bipolar disorder with high hydrophobicity, was first loaded in a high surface area mesoporous silica foam (MCF) with cellular pore morphology [59]. The aim was to enhance paliperidone solubility and simultaneously to prepare long active intractable microspheres. It was found that paliperidone, after its adsorption into MCF, was transformed in its amorphous state, thus leading to an enhanced in vitro dissolution profile. Furthermore, incorporation of the drug-loaded MCF into polymeric microparticles (PLA and PLGA) prolonged the release time of paliperidone from 10 to 15 days.

**Figure 5:**
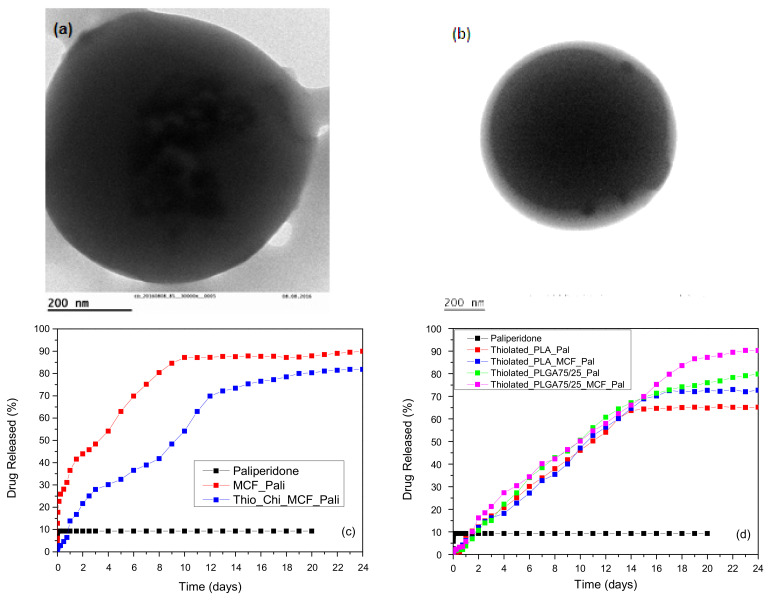
TEM images of microspheres: (**a**) Thiolated_PLA_MCF_Pal and (**b**) Thiolated_PLGA75/25_MCF_Pal and dissolution profile of (**c**) neat paliperidone, MCF loaded drug and thiolated CS microspheres, and (**d**) drug release profile from PLA- and PLGA-coated microspheres with thiolated chitosan [60].

In a similar study by Nanaki et al., a hybrid system for the intranasal delivery of paliperidone [60], was also studied. Paliperidone was first incorporated into MCF by adsorption, then encapsulated the MCF-drug system into PLA microspheres, which were eventually coated with thiolated chitosan. SEM images of Thiolated_PLA_MCF_Pal microparticles indicated that their sizes varied between 3–6 µm. TEM images also showed that paliperidone was detected inside the microspheres (Figure 5a) while a film on the surface of microparticles was formed from the thiolated chitosan (Figure 5b). Drug release studies showed that paliperidone is a hydrophobic drug with low solubility since only 10% of it was dissolved in the first hour without any further dissolution until 20 days (Figure 5c). When it is incorporated into MCF nanopores, the dissolution rate is substantially enhanced due to drug amorphization. Their addition to PLA and PLGA microparticles produces controlled released formulations up to 22–24 days (Figure 5d). However, the release rate is lower in PLA microspheres than PLGA due to the lower glass transition that PLGA has compared to PLA polymers.

Except for double emulsion techniques, other approaches have also been mentioned for the preparation of drug-loaded PLA MPs. In such an attempt, sustained release PLA microparticles of agomelatine drug have been prepared by using a solvent evaporation method combined with wet milling technology [61]. From dissolution experiments, it was found that agomelatine could be sustained release over the period of one month. The initial release mechanism was diffusion followed by erosion of the polymer matrix due to hydrolysis of PLA.

The rapid expansion of supercritical solutions (RESS) process has also been successfully employed by Vegara-Mendoza et al. to encapsulate coenzyme Q_10_ (coQ_10_), used for the prevention of cancer and neurodegenerative diseases, in PLA microcapsules [62]. Analysis of the effect of the PLA/coQ_10_ ratio and the cosolvent on microcapsule properties was conducted, indicating that both morphology and the size of microcapsules have been completely influenced by the PLA/coQ_10_ ratio. Similar shapes to the coQ_10_ have been obtained when the same concentrations were used. However, an increase in particle diameter was observed when the concentration of both materials was raised. Although the cosolvent in the supercritical system influences the particle size and the coQ_10_ solubility, it does not exhibit any effect on the morphology of the microcapsules or any interactions with PLA.

**Figure 6:**
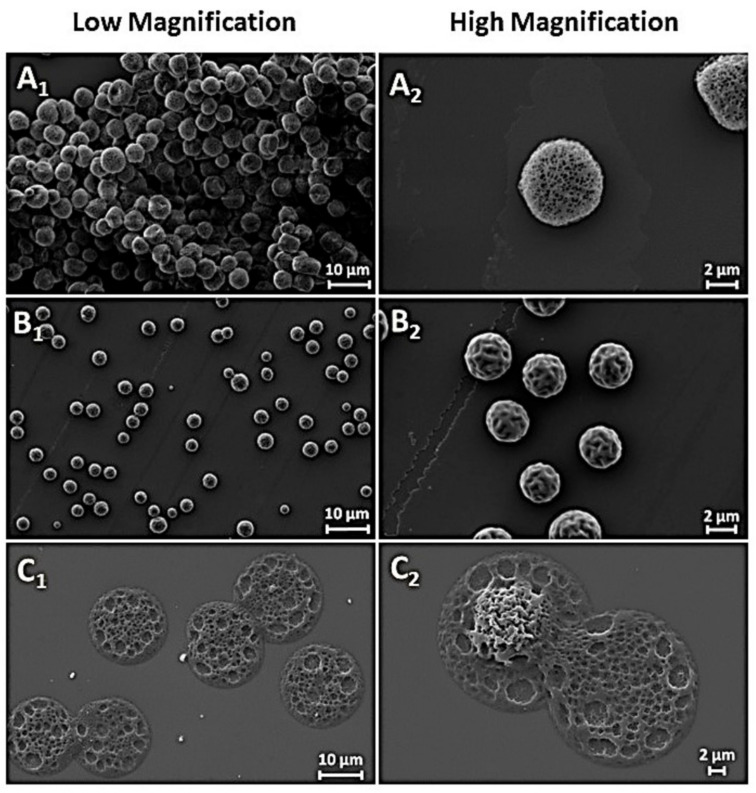
SEM images of (**A**) the 3 wt.% PLA–DCM group sample. (**B**) SEM images and of the 3 wt.% PLA ethanol–chloroform group sample. (**C**) SEM images of the 2 wt.% PLA–chloroform group sample [18].

Tasci et al. also worked beyond conventional methods of polymeric microparticle manufacturing in order to overcome some of their drawbacks, such as, for example, the time-consuming experimental procedures, among others. In their case, the electrospray method was used [18]. The formation of particles was performed using PLA solutions of three different organic solvents, namely dichloromethane, chloroform, and chloroform–ethanol mixture. The sizes and morphology of the prepared microparticles were optimized via well-controlled flow rates, applied voltages, and solvent concentration. It has been shown that DCM was the most suitable solvent for obtaining microparticles of a uniform spherical shape with an average diameter of 3.00 µm, and a highly porous structure (Figure 6).

**Figure 7:**
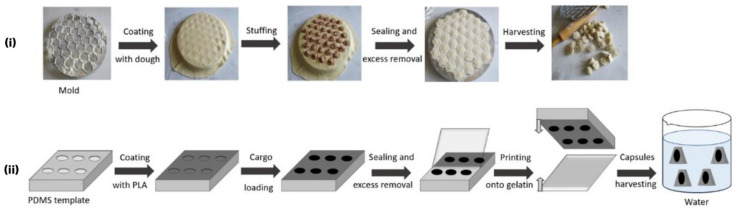
Schematic illustration of defined-shape microcapsules fabrication of (**i**) traditional pelmeni production process and (**ii**) PLA microcapsule harvesting in water [64].

With regard to shape control, the morphology of prepared PLGA/PLA has recently been compared using a microhomogenizer and a membrane emulsification technique [63]. It was found that the prepared particles were polydisperse and irregular in shapes using the classical microhomogenizer, while these prepared by membrane emulsification technique were very spherical and monodisperse. These differences also lead to completely different release profiles of the rifampicin drug, which is much faster in the first case and almost sustained and extended up to 14 days when the membrane emulsification technique was used. Likewise, Kudryavtseva et al. emphasized that customized shapes of microcapsules show clear advantages over spherical ones, having enhanced internalization by host cells, improved flow characteristics, and higher packing capacity [64]. A method for “defined-shape” polymer capsule fabrication aspired from the traditional pelmeni-dumplings from the cooking process was proposed (Figure 7).

**Figure 8:**
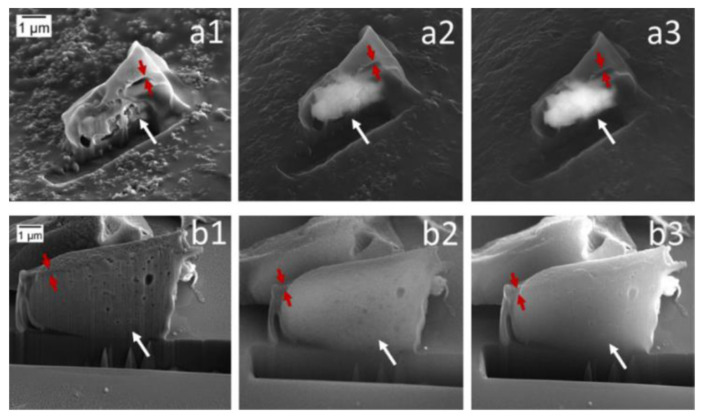
FIB-SEM cross-sectional image of capsules filled with (**a**) FeCl_2_ crystals and (**b**) Fe_3_O_4_ nanopowder obtained with (**1**) SE, (**2**) BSE detector, and (**3**) overlaid image. White arrows indicate the cargo crystals inside the capsules, while red arrows indicate the shell polymer layer [64].

PLA microcapsules of two different approaches, both having monodisperse size and shape distribution with about 7 μm long torpedo-like shape, were also fabricated. FeCl_2_ ground crystals, Fe_3_O_4_ nanopowder and carboxyfluorescein, were used as model cargoes for the prepared microcapsules (Figure 8). The study successfully demonstrated a well-defined core-shell structure, having high loading capacity, good cytocompatibility, and internalization by cells without causing toxic effects. Moreover, precise control over the microcapsule’s geometry has been successfully reached, providing significant flexibility for the choice of active cargoes, independent of their solubility and molecular weight. The microcapsule’s shell accordingly defines the capsule’s geometry, protects the cargo, and modulates its release, which may lead to a wide variety of different drug delivery strategies, whereas the co-encapsulation and surface modification can further facilitate their application according to targeted needs.

The shape/structure and size parameters are also very central in the work conducted by Ma’s research group toward the design of more stable DDS [65]. In their study, the strategies for maintaining the bioactivity of protein drugs during preparation and drug release have been analyzed and evaluated for future applications. First, the issue of controlling the size and uniformity of the microparticles was addressed. In this sense, a membrane emulsification process was developed by dissolving PLA on dichloromethane, acting as the dispersed phase, with the continuous phase being water containing poly(vinyl alcohol) (PVA) and sulfate. The diameter by direct membrane emulsification process could be effectively controlled from submicron to 100 μm, and size distribution (CV value) was estimated at around 10%. Maintaining the bioactivity of the encapsulated molecules by stabilizing their 3 and 4 dimensional structures was then a further challenge. For that, several different strategies were followed, such as adding additives into protein solution, using solid drug powder instead of protein solution, a rapid self-solidification process, a step-wise crosslinking process, and dispersing drug powder in oil phase to decrease the contact of protein molecules and hydrophobic interface, to mention a few. In addition, it was concluded that the mild membrane emulsification process does not create high shear forces capable of altering the protein structure. The addition of stabilizing substances, such as HP-β-CD (hydroxypropyl-β-cyclodextrin), PVA, and PVP (polyvinylpyrrolidone), in the inner water phase can maintain bioactivity satisfactorily. The resulting PLA microspheres were able to maintain the bioactivity of the encapsulated proteins and sustain their release for about a month. Such microspheres can be used to carry protein molecules, such as insulin, recombinant human growth hormone (rhGH), and other similar molecules.

Working also with biomolecules, Icart et al. focused on the microencapsulation of glucagon-like peptide-1 (GLP1) in PLA matrices using the double emulsion–solvent evaporation technique, where particles were formed by solvent evaporation under constant stirring and were collected through centrifugation [66]. Glucagon-like peptide-1 (GLP1) is a naturally occurring peptide used in cardiovascular and weight loss applications; however, its clinical application is still limited due to a half-life of 2 min as a result of rapid enzymic degradation. The results of the study showed that PLA had a determining effect on the peptide in in vitro release profile. More specifically, the release occurred in three phases, starting with an initial burst release of surface drug concentration, followed by a slow-release rate until the polymeric microparticle matrix was degraded, resulting in an accelerated-release phase by the end of the degradation process that lasted for a total of 25 days. This in vitro case study was further backed up with evidence through in vivo experiments, and it strongly suggests that hGLP1-loaded PLA microparticles can provide sustained drug release for weeks and be potentially useful for clinical applications.

Recent developments in stem cell-based therapy methods have utilized the dual microencapsulation of stem cells, targeting bone repair by introducing bone marrow mesenchymal stem cells (BM-MSCs) and bone morphogenetic protein-2 (BMP-2) to the affected area in order to repair large bone defects. The research of Kong et al. [67] describes the formation of a multicore microcapsule with the internal core, enclosing BM-MSCs, made of sodium alginate and the outer shell made of PLA with encapsulated BMP-2. The microcapsules were formed through electrospraying. The sustained release of the combination of stem cells and proteins for 30 days in vitro encouraged Kong et al. to progress to the in vivo treatment of bone structures in rats. The transplantation of the microcapsules into the affected tissues led to sufficient repair of the bone after 4 to 8 weeks. Thus, the research reached the conclusion that the transplantation of BM-MSCs and BMP-2 in multicore PLA microcapsules is a promising strategy for regenerative therapies of large bone defects.

Lastly, long-acting injectable (LAI) microspheres are a fascinating category of formulations, functioning as depot systems for effective drug administration, providing enhanced stability and bioavailability, improved efficiency, and patient compliance. These systems are also appropriate for encapsulation of sensitive active agents, such as peptides and proteins. According to Butreddy et al. in their relevant review article [68], PLA/PLGA-based LAI microspheres can have a really positive impact on the delivery of proteins/peptides. They are able to deliver drugs to targeted areas, achieving higher drug concentrations on-spot and reduced systemic exposure. The LAI microspheres must have uniform size distribution at large scale production and must retain consistent bioactivity of the encapsulated drug during preparation, storage, and release. Emulsion–solvent evaporation, coacervation, and spray drying are three important manufacturing techniques, with emulsion–solvent evaporation being currently the most reliable one in clinical LAI microspheres preparation.

### 4.2. PLGA Microparticles

The combination of lactic acid (LA) with glycolic acid (GA) results in a copolymer system known as PLGA, a very attractive and powerful option and one of the most established ones in the pharmaceutical field. PLGA systems are comprehensively examined as sustainable drug delivery systems due to their biodegradability, biocompatibility, morphology, particle size, and sustained drug release properties in various in vivo and in vitro systems. Its polymeric properties, such as its glass transition temperature (Tg) and degradation rate, can also be fine-tuned by modifying the LA:GA content ratio [27]. At the moment, approximately 20 PLGA-based products are commercially available with the approval of the US Food and Drug Administration FDA and the European Medicines Agency EMA and are used as delivery vehicles for drugs, proteins, and numerous macromolecules in therapeutic applications [69,70].

**Figure 9:**
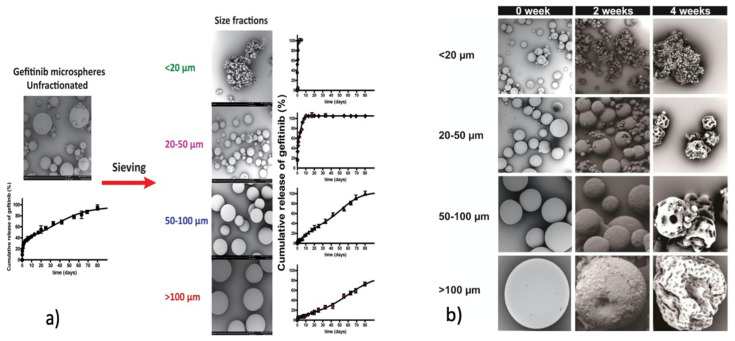
(**a**) Effect of particle size on in vitro release of gefitinib-loaded PLGA microspheres. (**b**) SEM micrographs of gefitinib-loaded PLGA microspheres upon in vitro incubation in buffer of pH 7.4 and at 37 °C. Adapted with permission from ref. [71]. 2017, ACS Publications.

PLGA microspheres loaded with the gefitinib drug were prepared using an oil-in-water solvent evaporation method, obtaining different particle sizes of 5 ± 1, 32 ± 4, 70 ± 3, and 130 ± 7 μm [71]. Encapsulation efficiency of gefitinib, loading content, and microspheres yields are all increasing by increasing the particle size of prepared microspheres. In vitro drug release studies showed that microspheres with sizes smaller than 50 μm have a rapid diffusion-based release, reaching completion within 2 days. For such low particle sizes, their high surface area per unit volume leads to a higher rate of water permeation and thus to higher matrix degradation rates (Figure 9a). Larger microspheres, however, showed a sigmoidal release pattern that continued for three months in which diffusion (early stage), as well as particle erosion (later stage) governed drug release (Figure 9b). Therefore, it is clear that the two main mechanisms that drive drug release from PLGA microspheres are diffusion and degradation/erosion [72].

Gangapurwala et al. examined the potential use of supercritical CO_2_ fluid (SC-CO_2_) for the fabrication of PLGA microparticles for drug delivery systems. Its tunable properties above critical temperature and pressure provide control of the particle size, particle morphology, and drug loading [27].

A combination of various highly porous PLGA MPs supported by an in silico non-linear first-order model in order to predict and obtain transitional situations and tunable release of curcumin, while guaranteeing extended or rapid drug release [73]. Three different configurations were obtained (CUR–NE, CUR–oil, and CUR–water) in order to produce microspheres with curcumin molecules embedded inside or outside the porous structures. This approach can be applied to other molecules and drugs, giving the option of avoiding additional experiments. In that way, the drug release will occur with a controlled timing, in a tunable amount, optimizing the therapeutic effectiveness and, therefore, decreasing potential side effects.

In another study, Molavi et al. incorporated nucleophilic (risperidone) and basic (olanzapine) drugs into PLGA microspheres [13]. The results demonstrated significantly high polymer degradation, while the release profile of risperidone was biphasic, and in contrast, the Mw of the placebo microspheres was completely unchanged. Furthermore, the results and rapid initial release were attributed to the reaction of the weak basic drug and the acidic polymer. A plateau was reached through hydrolysis of produced acidic monomers after those reactions of neutralization of basic drugs took place. These findings were proven at high risperidone and olanzapine loadings.

Ding et al. prepared chitosan-coated PLGA microparticles with controlled diameters from 5 to 120 μm using capillary microfluidic techniques for ocular drug delivery systems [74]. Severe ocular environments highlight the necessity for mucoadhesive micro-particulates with the ability to survive such harsh conditions. Furthermore, studies have shown that the presence of such particles is prolonged on corneal surfaces, and thus, the bioavailability of drug systems topically applied to the eyes is significantly enhanced.

**Figure 10:**
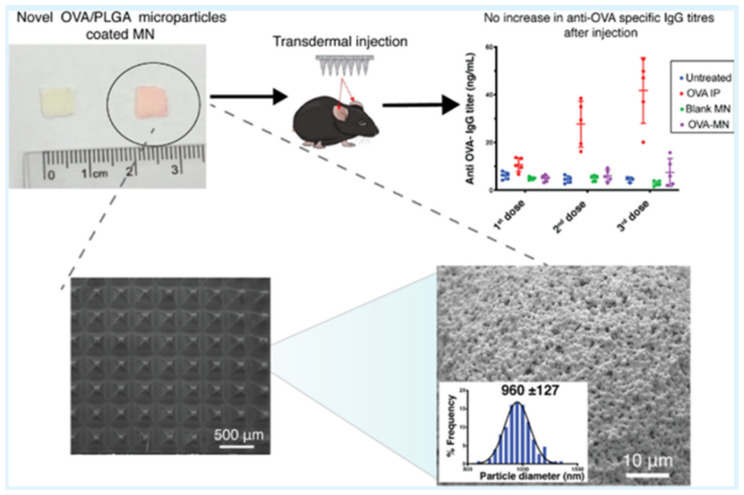
Novel OVA/PLGA coated MN microparticles for transdermal application, with the particle size distribution. Adapted with permission from ref. [75]. 2020, ACS Publications.

PLGA microparticles loaded with Ovalbumin (OVA), a protein traced mainly in eggs, were fabricated onto hydrogel-forming microneedle arrays with the use of electrohydrodynamic atomization (EHDA) by Angkawinitwong et al. [75] (Figure 10). An extended release of ovalbumin over ca. 28 days was subsequently recorded, while similar mechanical characteristics and insertion properties to the uncoated system were manifested by the particles. Thus, the possibility of using EHDA to coat a microneedle array seems very prospective as a novel noninvasive protein delivery strategy for transdermal applications.

Concerning the administration of biologics, parenteral routes, such as intravenous and/or intramuscular injections, have been mostly used due to their low oral bioavailability and stability in the gastrointestinal tract, which can negatively affect patient compliance. As an alternative non-invasive route for the administration of pharmaceutical ingredients, recently pharmaceutical researchers have focused their studies on pulmonary delivery [76]. The pulmonary route is an attractive target for both local and systemic drug delivery due to benefits, such as rich blood supply, large surface area, and absence of first-pass metabolism. Inhalable PLGA MPs have been widely studied in an attempt to find strategies for prolonged release of drugs in the lung [35] (Table 2). The small size of the inhalable particles is highly necessary for appropriate lung deposition but may result in low drug encapsulation into MPs. After the drug is administrated, its dispersal in the lung and preservation in the favorable site of the lung is significant for the treatment to be effective.

Chlorhexidine diacetate (CDA) and digluconate (CDG) are the most frequent salts used in dental care. PLGA microparticles containing CDA and CDG were prepared successfully by Sousa et al. [86]. PLGA microparticles containing diacetate salt demonstrated a viable and homogeneous drug release over 120 days, while digluconate salt solution showed a more immediate release and neither is subject to thermal degradation. Both MPs had an antimicrobial effect against *Streptococcus mutans*, and their drug release profiles demonstrated the systems’ suitability to control these bacteria in the oral environment using PLGA MPs containing CDA or CDG. Although it is very efficient in the control of this microorganism, it presents some disadvantages, such as tongue discoloration, taste alterations, and teeth restoration staining. PLGA microparticles containing CDA proved to be feasible and could be incorporated in temporary restorative dental materials.

**Figure 11:**
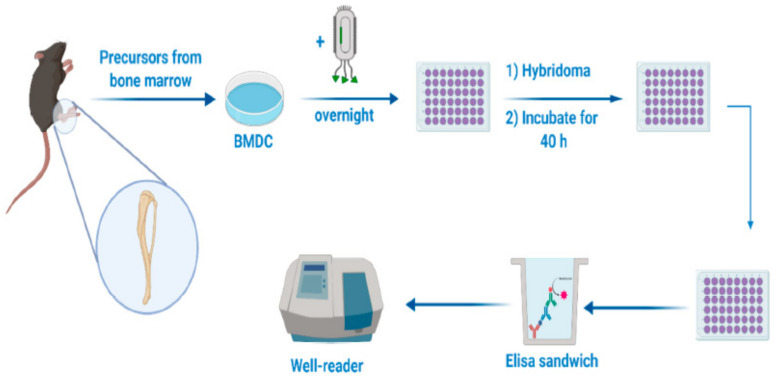
Dendritic cells (DCs) were incubated with bacteriophage overnight. B3Z hybridoma cells were added, and cells were co-cultured for 40 h. Later, sandwich ELISA was conducted to evaluate Interleukin-2 in supernatants [2].

Jamaledin et al. proposed the use of MPs made of PLGA to encapsulate fd bacteriophage for the first time. For bacteriophage–PLGA MP synthesis, the water in oil in water (w_1_/o/w_2_) emulsion technique was used (Figure 11). The immunogenicity of the encapsulated bacteriophage after being released by MPs was demonstrated using recombinant filamentous bacteriophages expressing the ovalbumin (OVA) antigenic determinant. Their results revealed that encapsulated bacteriophages remained stable and maintained their immunogenic properties [2].

Artemether, a highly efficient antimalarial drug, possesses the potential to treat patients tormented by *Plasmodium falciparum* (P.F). However, poor therapeutic effects can be caused by the fact that artemether may be degraded rapidly by stomach acids and cleared quickly from the body after oral administration. Aiming to solve this problem, artemether is combined with piperine (AP), a naturally occurring alkaloid and excellent bio-enhancer, which can enhance the bioavailability of numerous drugs and other phytochemicals. Ali et al. fabricated two types of core-shell MPs based on PLGA and chitosan (CS) by using a coaxial electrospray system (CES) loaded with both artemether and piperine to achieve a sustained drug release [87]. The PLGA or PLGA-CS shell caused improved sustained drug release behavior in both types of MPs. The shell protected the fast degradation of artemether caused by acidic gastric juice. The results indicated that AP-PLGA-CS and AP-PLGA microparticles embrace the potential within the application of malaria treatment and provide a promising platform to encapsulate multiple drugs in polymeric particles for drug delivery.

Multiple drugs have also been microencapsulated in PLGA scaffolds by Aina et al. [88]. Specifically, Metronidazole, Paracetamol, and Sulphapyridine were encapsulated into PLGA scaffolds and then studied using the X-Ray Powder Diffraction (XRPD) technique. Changes in the diffraction patterns of those scaffolds after encapsulation suggested a chemical interaction between the pure drugs and the scaffolds. The drugs can be encapsulated in the scaffolds even at low concentrations, as permitted by their aqueous solubilities. No physical intermixture was observed.

Very recently, Kulkarni et al. aimed to create a drug delivery and release system that may be helpful as a novel therapeutic and anticancer system to slow down the development of diseases that are associated with oxidative stress [89]. Corn silk contains flavonoids that contribute to its antioxidant and anticancer activity. An efficient delivery system was fabricated using the solvent extraction method to incorporate anticancer methanolic corn silk extract. Spherical and relatively small (d = 485.9) polymeric microparticles were obtained containing flavonoids with encapsulation efficiency (EE) of 60.66%. MTT cell viability assay was performed on HeLa, NIH 3T3 cell lines, and the cellular uptake of the drug was studied using fluorescence microscopy, confirming the uptake by the cells within 24 h of treatment. The MPs were proven non-toxic to normal cells, and the system provided protection and controlled release of the bioactive compounds.

**Figure 12:**
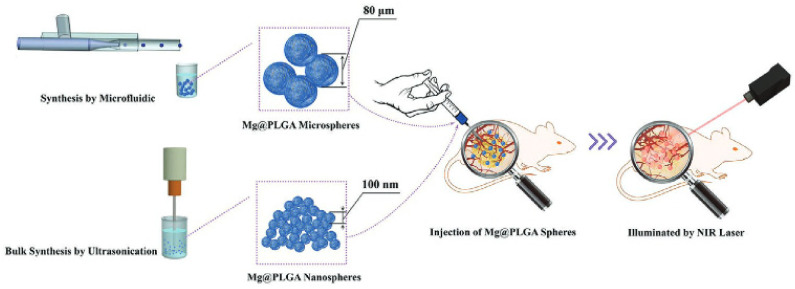
Schematic illustration of microfluidic and bulk methods for fabrication Mg@PLGA microspheres and nanospheres for photothermal tumor treatments in vitro and in vivo. Adapted with permission from ref. [90]. 2021, John Wiley and Sons.

Zhou et al. investigated the controlling factors for cytotoxicity, photothermal, and anti-tumor effects of biodegradable magnesium poly(lactic-co-glycolic acid) (Figure 12), in vitro and in vivo. MgPLGA microspheres were made by microfluidic emulsification and demonstrated high Mg encapsulation efficiency, 87%. The photothermal and anti-tumor effects of MgPLGA spheres were determined by their Mg content, irrelevant to their structural features and size, as shown in in vitro cell assays and in vivo mice models. These results provided important implications for designing and fabricating stimuli-responsive drug delivery vehicles [90].

Subha et al. used beeswax and PLGA as wall materials in order to obtain sustained and controlled release of Capecitabine, an anticancer drug [91]. First, the beeswax microspheres loaded with the drug were formed using the melt dispersion technique, and then were dispersed within the drug-incorporated PLGA. Consequently, the beeswax microspheres create the inner core of the drug delivery system. The beeswax vehicles were designed via the coacervation and phase separation method and had a diameter of 3441 nm. The drug release from the microcapsules was tested in simulated gastric fluid, and a slow and controlled release of the drug was observed (PH 6.8 at 37 °C). Approximately 25% of the drug was released in 24 h, concluding that beeswax microspheres could be considered as potential drug delivery vehicles and drug targeting in cancer therapy.

**Figure 13:**
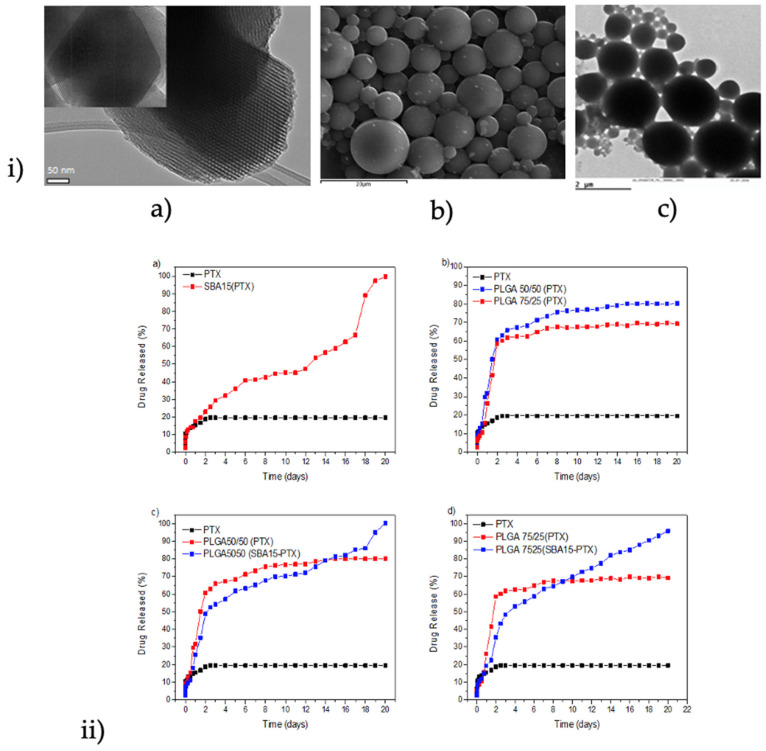
(**i**): (**a**) TEM micrographs of the parent SBA-15 mesoporous silica (**b**) SEM micrographs of PLGA 50/50 *w/w* microparticles (**c**) TEM micrographs of PLGA 50/50 *w/w* microparticles loaded with PTX/SBA-15, low magnification (**ii**) in vitro release of PTX (**a**) from SBA-15, (**b**) PLGA 50/50 and 75/25 *w/w*, (**c**) PLGA 50/50 *w/w*, PLGA 50/50 *w/w* loaded PTX/SBA-15, and (**d**) PLGA 75/25 *w/w*, PLGA 75/25 *w/w* loaded PTX/SBA-15. Adapted with permission from ref. [92]. 2021, Elsevier.

In a recent study, SBA-15 mesoporous silica was loaded with paclitaxel (PTX) anticancer drug and was then encapsulated in two different copolymers of PLGA (50/50 and 75/25 *w/w*), forming composite microspheres appropriate for topical injection [92]. The target was to increase the drug loading capability and to enhance its solubility. The TEM micrographs shown in Figure 13(ia) verify the well-formed structure of the SBA-15 mesoporous silica that was used in this study, which was loaded with PTX and encapsulated in PLGA microparticles, which have spherical morphology with sizes of 8–12 μm (Figure 13(ib)). It was found that the molar ratio of LA/GA in copolymers (i.e., PLGA 50/50 and 75/25 *w/w*) have no significant effect on the sizes of prepared microspheres. A more precise observation in Figure 13(ic) shows that PTX/SBA-15 was successfully embedded into the microparticles and can be observed as black shadows in the respective images.

From dissolution profiles, it is clear that neat PTX has a very low solubility since even after 12 days in SBF medium its dissolution is less than 20% (Figure 13(iia)), while when adsorbed onto SBA-15, its solubility was significantly improved since the whole amount of PTX was dissolved during day 12. This behavior is associated with the amorphization of PTX, as was proved by DSC and XRD studies. An enhancement was also observed when PTX was encapsulated in PLGA microspheres (Figure 13(iib)). The PLGA copolymer slightly affected the release rate, which is higher in PLGA 50/50 than PLGA 75/25 *w/w*, due to the lower Tg of the first copolymer, as well as due to the high drug loading. A multiphasic release was observed from both copolymers with an initial burst release for the first 1–2 days, followed by a slower sustained release up to 3 weeks. This behavior was changed when the SBA-15 loaded with PTX was microencapsulated into the PLGA matrix (Figure 13(iic,d)). The burst release noticed in the first 2 days is lower due to the slower diffusion rate of PTX from SBA-15. Additionally, the release rate from both composites is more sustained compared with neat copolymers.

### 4.3. Microparticles Based on Amphiphilic PLA/PEG and PLGA/PEG Copolymers

**Figure 14:**
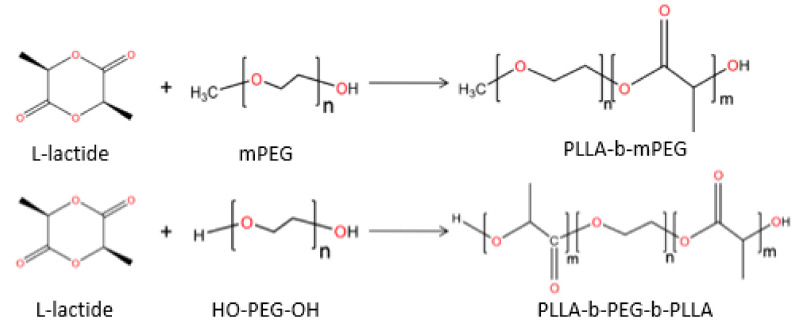
Synthesis of PPLA/PEG star-shaped copolymers by ring opening polymerization.

Amphiphilic biodegradable and bioresorbable microparticles consisting of hydrophilic-hydrophobic parts have gained significant attention across multiple fields of biochemistry for direct drug delivery because of their useful rheological, biomedical, and mechanical properties. Amphiphilic copolymers tend to self-assemble into core-shell micelles with a hydrophobic center and a hydrophilic external surface in aqueous solution. PLA’s high hydrophobicity makes it an excellent biomaterial for such applications, while poly(ethylene glycol) (PEG) is a biocompatible hydrophilic polymer used extensively in many applications in pharmaceutical technology. Such poly(l-lactide)/poly(ethylene glycol) (PLLA/PEG) copolymers can be easily prepared by ring opening polymerization of lactide in the presence of PEG and a catalyst, usually stannous octoate [93]. Hydroxyl end groups can act as initiators for lactide polymerization, and according to this procedure diblock or triblock copolymers can be prepared. Ding et al. synthesized such amphiphilic PLLA/PEG copolymers (Figure 14) with various molecular weights for the delivery of ibuprofen (IBU), used for the treatment of a range of aches and pains [94]. Studies showed that by increasing the Mw of PEG, the encapsulation efficiency and drug loading content and of IBU were improved significantly in the PLLA-PEG micelles. Another interesting application of PLA-PEG diblock copolymer is the treatment of retinal diseases. Rafat et al. [95] encapsulated the Tat-EGFP protein (Tat; protein transduction of HIV-1 trans activator of transcription protein), which can effectively circumvent the retina’s barriers. Four different ratios of PEG–PLA microparticles were developed by changing the ratio of the protein and the polymer. The microcapsules exhibited a burst release profile in the first days. As the polymer concentration was increased to a constant protein concentration, the release rate was reduced.

Similar amphiphilic copolymers can be prepared by using PLGA instead of PLA. Aspirin from primary times of marketing approval became a drug effective for common inflammations, fever, and pain reduction. Liu et al. [96] loaded aspirin in PLGA-PEG-PLGA copolymers while additionally checking the impact of including organic montmorillonite (o-MMT) in microspheres. MPs containing higher concentrations of montmorillonite show a faster rate of drug release despite the irregular effects of the first few hours. Compared to copolymer microspheres, microspheres with o-MMT gave a better release profile and are recommended for research. The ABA tri-block copolymer (PLGA-PEG-PLGA) was used by Khodaverdi et al. [91] for insulin microencapsulation. Microspheres were created by the microwave heating technique without the use of organic solvents throughout the preparation. They observed regulated drug release for up to 3 weeks, at which point the polymeric matrix may have begun to degrade. However, in the case of encapsulation of very small molecules, such as clonidine, the choice of PLGA-PEG copolymers according to Gaignaux et al. [97] will not give satisfactory encapsulation results, as due to their high porosity, molecules can easily penetrate the hydrophilic network.

**Figure 15:**
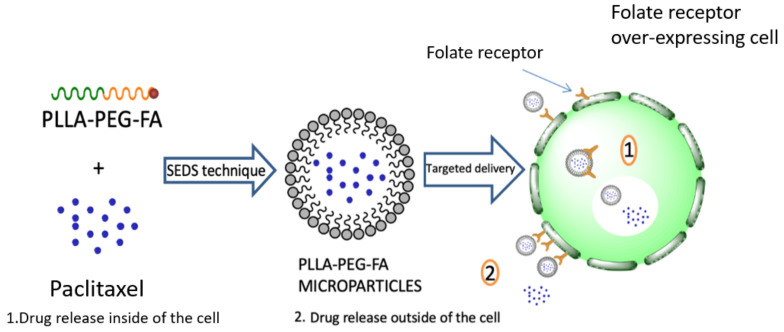
A scheme of folate receptor mediated target delivery of PTX using FA-PEG-PLLA copolymer microparticles.

Another interesting application of these copolymers is for cancer treatment. PEG can be used to modify microparticle surfaces in order to avoid their recognition by cells of the mononuclear phagocyte system, thus leading to an increased blood circulation time of microparticles [98]. These PLA/PEG amphiphilic carriers exhibit prolonged blood residence after their administration (long-circulating drug carriers), as well as passive targeting of tumors due to the leaky vasculature of many tumors and the ‘enhanced permeability and retention’ (EPR) effect [99,100]. Nevertheless, nanoparticles prepared from copolymers with high molecular weight PLA blocks exhibited longer blood circulation times after i.v. administration in rats than the nanoparticles prepared from copolymers with low molecular weight PLA blocks [101] and have been used extensively as drug delivery Microparticles (Table 3). Additionally, from in vivo studies performed in mice, it was found that blood circulation time could be increased by increasing the molecular weight of the PEG blocks in diblock PEG-PLGA copolymers, from 5000 to 20,000 g/mol [102]. These copolymers have long been established as ideal carriers for anticancer drugs [103,104,105]. Despite the generally positive outcomes, therapeutic agents have several drawbacks, including weak pharmacokinetics, unspecific bio-distribution, and limited targeting efficiency. When therapeutic agents are used in cancer treatment, low solubility and hydrophobicity are considered significant obstacles [106]. To overcome them, anticancer drugs, such as paclitaxel and doxorubicin, are encapsulated in amphiphilic copolymer micelles, such as PEG/PLLA. Huang et al. [107] used folic acid (FA) in conjunction with PEG terminal groups to form FA-PEG-PLLA for tumor targeted therapy (Figure 15). Owing to the overexpression of FA receptors in many cancer cell types, folic acid is one of the most commonly used targeting ligands [108]. Microparticles loaded with paclitaxel (PTX) were prepared using the solution-enhanced dispersion by supercritical fluids (SEDS) technique with CO_2_ used as the supercritical fluid. The diameters of most of the particles were 1–3 μm, with a spherical shape and slight agglomeration.

Ouyang et al. [109] fabricated a similar system. PLA-PEG-PLLA triblock copolymer was synthesized via the SEDS technique with different PEG ratios (0, 5, 10, 15, 25, 50% *w/w*). Results resembling Huang’s previous work [107] were obtained when a PEG content of 25% (*w/w*) was used. Lumpy grains were grouped together, making them unsuitable for use as drug carriers. A PEG content increase from 10 to 15% *w/w* causes crystallization of paclitaxel outside the microspheres. Further increase of PEG content in the copolymer matrix decreases the drug loading, as well as the encapsulation efficiency of the developed microparticles.

SEDS process was also used to create microspheres from a morphine-loaded PLLA-PEG-PLLA triblock copolymer. Morphine is a naturally occurring analgesic drug used for the relief of cancer pain. The work of Chen et al. [110] showed that the rate of encapsulation and release of the drug are a function of the percentage of poly(ethylene glycol) groups. The results indicate that triple block with 3% PEG ratio shows a better profile as it brings 80% release of morphine in 48 h.

PTX was loaded into magnetic microspheres prepared by PLA-PEG copolymer with magnetite [111]. The microspheres were created by the solvent evaporation method with an average diameter of 21.73 ± 12.40 μm. The drug was released at a high rate in the first 5 days, with or without the application of an electric field, and was sustained for the upcoming periods under the application of a magnetic field. The goal of Ruan et al. [112] was to show that the PEG-PLA copolymer was effective against hydrophobic PLGA. A controlled release through carriers for no more than one month is required for an anticancer drug. It was concluded from the study that the release is more satisfactory in the case of the PLA-PEG-PLA copolymer, which is also affected by the presence of acetone.

5-Fluorouracil (5-FU) in combination with microspheres from mPEG-PLA was tested as an anticancer therapy by Xiong et al. [113]. Increasing the amount of diblock copolymer led to an increase in the rate of encapsulation of the drug in the microspheres, as due to the higher viscosity, the migration of the drug was avoided. However, increasing the amount of mPEG in the copolymer, on the other hand, had no such impact. The release of the drug was quite large in the first hours, as expected.

In addition to drugs, other naturally occurring substances, such as perillyl alcohol (POH), a phenolic monoterpene, have been shown to block tumor cell cycles by inhibiting their migration. Its potential use in the treatment of glioma is indeed promising [114]. Marson et al. [115] worked with poly(d,l-lactic acid)-block-poly(ethylene glycol) (PLA-b-PEG) polymer-based carriers as a delivery platform for POH. Microcapsules were prepared by the solvent evaporation method with two different tensoactives: PVA and sodium cholate (SC). Both samples presented similar release profiles, displaying complete drug unloading after 3 h.

Another significant goal of the medical community is to develop myocardial regeneration systems for the treatment of myocardial infarction. The contribution of PLGA copolymers to this research has been demonstrated by Pascual-Gil and co-workers [117]. They prepared MPs from PLGA in combination with PEG to avoid mainly opsonization in blood, and the growth of factor-loaded particles, neuregulin (NRG), was administered to rats. PEGylation led to an increase in the retention of microparticles in the myocardium up to 12 weeks. A similar tactic was followed by Kirby et al. [118] for bone regeneration. In this case, bone morphogenetic protein 2 (BMP-2) was used as the growth factor. The triblock PLGA-PEG-PLGA acts as a plasticizer, while the hydrophilic environment that is created increases the hydrolysis of PLGA and at the same time the release of drug until day 10. Lysozyme was used by Tran et al. [119] as the original protein, which exhibits behaviors similar to growth factors for tissue engineering. Due to the high cost of the growth factors, a polymer system is required to have a good release profile while loaded with a small amount of them. This work is also an indication of the large contribution of PEG groups, as a release of up to 58% was observed until the 8th day in the triblock with PLGA20PEG20 ratio.

Furthermore, Li et al. [116] suggested the triblock copolymer poly(lactic acid)-poly(ethylene glycol)-poly(lactic acid) (PLA-PEG-PLA), PELA for bone regeneration. Bone morphogenetic protein (BMP) was encapsulated in the copolymer in microspheres. The maximum EE (%) was obtained when the Mw of PEG was 4000 Da (27.6%) due to the hydrophophilicity increase. EE also reached its highest value (26%) when the amount of PELA was 330 mg. All these applications have been summarized on Table 4.

### 4.4. PLCL Microparticles

Poly(ε-caprolactone) is a semicrystalline polymer with rubbery properties that exhibits good biodegradability, biocompatibility, and permeation to drugs and thus has attracted great attention for medical applications [120]. Copolymers of l-lactide and ε-caprolactone (PLCL), offer great permeability, better efficiency of degradation rate, thermal and mechanical features that can enhance their processing, provide long-term delivery, and present great interest for immunosuppressive drugs, as slowly degradable materials. Materials obtained from poly(ε-caprolactone) undergo slower degradation than PLGA, and poly(d,l-lactide) PDLA and may be used in systems that provide drug delivery even extending over a period of more than one year [120,121]. For instance, copolymers from ε-CL and l-LA were reported to degrade in vitro at a rate dependent on the l-LA content and PCL crystallinity [122]. Their PLCL copolymer is a promising replacement for medical applications because of its controllable elasticity and the capacity to change the ε-caprolactone/l-lactic molar ratios and their mechanical properties [120]. Degradable microspheres have gained attention as delivery vehicles for steroids in postmenopausal therapy. Copolymers of ε-CL and d,l-LA have been used to prepare microspheres for prolonged release of progesterone and b-estradiol. The system offered a constant release for up to 40 days in vitro and 70 days in vivo [122].

At a glance, Hitzman et al. developed a respirable microcarrier based on poly-(lactide-co-caprolactone) (PLCL) using a spray drying technique for sustained release of 5-fluorouracil (5-FU), which has been extensively studied as a chemotherapeutic agent [123]. Microdialysis was used to determine the release rate of 5-fluorouracil from liposomes, microspheres, and lipid-coated nanoparticles (LNPs) and to study their use as a respirable delivery system therapy of lung cancer for adjuvant (post-surgery). Microspheres may deliver high concentrations of drug for an extended time by controlling their size and porosity. Different systems based on PLCL and PLGA microspheres were compared, and the results showed that PLCL microspheres released 5-FU faster compared with PLGA systems.

**Figure 16:**
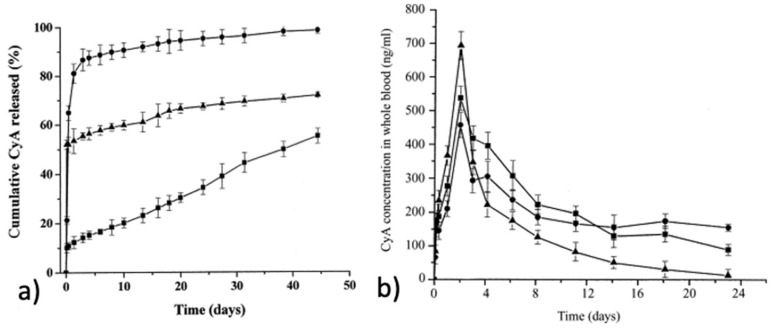
(**a**) In vitro release profiles of cyclosporin A-loaded microspheres in 0.1 M phosphate buffer solution (pH 7.4, containing 0.2% SDS) at 37 °C. (●) CyA-P(LA-b-CL) (48.1/51.9); (▴) CyA-P(LA-b-CL) (78.7/21.3) and (■) CyA-PLGA (80/20). (**b**) In vivo release profiles of cyclosporin A-loaded microspheres (suspended in 1 mL of 0.1% CMC-Na) after subcutaneously injected into Wistar rats. (▴) CyA suspension; (■) CyA-P(LA-b-CL) (48.1/51.9) and (●) CyA-P(LA-b-CL) (78.7/21.3). Each point represents the mean ± S.D. of four animals. Adapted with permission from ref. [124]. 2021, Elsevier.

Copolymers from ε-CL and l-LA can also be interesting in developing alternative release systems of cyclosporine A (CyA) and rapamycine (sirolimus), in which available dosage forms cause a lot of side effects [121]. Li et al. carried out in vitro and in vivo studies about microspheres, loaded with cyclosporin A, based on copolymers of lactide and ɛ-caprolactone [123]. Cyclosporin A (CyA), a hydrophobic peptide, was incorporated in microspheres based on poly(lactide-b-caprolactone) (P(LA-b-CL), LA/CL (in molar ratio): 78.7/21.3 and 48.1/51.9) and poly(lactide-co-glycolide) (PLGA, LA/GA: 80/20) using the oil-in-water (O/W) emulsion solvent evaporation method. It appeared that CyA can be efficiently incorporated in the microspheres (exceed 96%). Compared with PLGA microspheres, P(LA-b-CL) microspheres released CyA faster (Figure 16a), which can be attributed to the partial crystallization occurring in P(LA-b-CL) microspheres. CyA levels in whole blood were also examined. Compared with PLGA microspheres, poly(lactide-b-caprolactone) microspheres provided a higher blood level of CyA (Figure 16b). In all cases, CyA release can be divided into two different phases: burst release within the first few days and the subsequent sustained release.

A one-week subdermal delivery system for l-methadone was developed by Cha et al. [125]. Microspheres containing 13–16% l-methadone were prepared from three biodegradable polymers, PLLA, PGLA, and poly(ε-caprolactone-co-l-lactic acid) (PCL-L,LA), using the solvent evaporation method. L-methadone’s release from PCL-LA microspheres (75–85 mol% l-lactic acid) was completed within 48 h. PGLA (80 mol% l-lactic acid) microspheres showed almost as fast release, but 20% of the drug remained in the polymer matrix. Release from PLLA microspheres was subject to a 3–4 day induction period prior to loss of the drug during the next five days. This induction period for PLLA and the direct release of l-methadone from PGLA microspheres were the consequences of an exceptionally large acceleration of the hydrolytic chain cleavage of the polymers in the presence of the basic drug.

Microspheres from the copolymers of lactide and ε-caprolactone find an interesting application in the controlled release of steroids, such as progesterone and b-estradiol [126]. These copolymers contained 83–94% of L or d,l-lactide. The influence of the microstructure of lactidyl blocks in the copolymer chains on the drug release rate has been investigated. A more uniform release rate appeared in copolymers derived from d,l-lactide as composed of L-lactide. For the copolymer containing 83–94% of d,l-lactide units, the progesterone and b-estradiol release rate in vitro was found to be practically constant over 40 days. The in vivo studies performed on rats revealed that the period of constant release rate of b-estradiol could be prolonged to about 70 days.

Microspheres of poly(dl-lactide-co-caprolactone) (86 mol% dl-lactide) have also been prepared by Kassab et al. in order to incorporate into them nystatin, an antifungal drug [127]. Microspheres were prepared using the oil-in-water (o/w) emulsion solvent evaporation technique. The percentage yield was high, and the drug entrapment efficiency for the dimer depended on the quantity of nystatin in the formulation, while the microspheres were spherical in shape with an average size between 80 and 110 μm. The release profile was slow during the first week, then rapid during the second week to reach a maximum close to 90% for the formulation that contained the highest quantity of nystatin. Moreover, the microspheres were stable, and no degradation was observed during the period of study of two months.

Zhu et al. encapsulated ibuprofen in PLCL microspheres (LA/CL: 78.7/21.3 by mole), a non-steroid drug commonly used in the treatment of post-operative, epidural, arthritis, dysmenorrhea, and dental pain [127]. For the preparation of microspheres, the oil-in-water (o/w) solvent evaporation method was used. The results indicated that the drug entrapment efficiency was about 80%. The complete ibuprofen release duration from the microspheres exceeded 1 month. The results showed that ibuprofen was partially crystalline in PLCL microspheres. The results suggested that the copolymer could have potential applications for long-term ibuprofen release. The use of PLA-PCL copolymers for drug microencapsulation is summarized in Table 5.

### 4.5. Formulations Based on Star-Shaped PLA or PLGA

Several researchers concluded that star-shaped PLA (s-PLA) presents better rheological and mechanical properties than linear PLA [129]. s-PLA has a compact structure with a smaller hydrodynamic radius, lower solution viscosity, increased strength, and peculiar morphologies compared to linear PLA with a similar molecular weight [130,131,132]. These features make s-PLA difficult to get stranded in blood and ensure the bioavailability of the encapsulated drug [133]. Moreover, s-PLA has attracted considerable attention due to its special three-dimensional structures, containing more end-groups that may be treated as functional groups. s-PLA can connect more effectively with targeted molecules, which contribute to the transport of encapsulated drugs to the targeted organs and tissues [133,134]. The features of synthesized s-PLA could modify the pharmacokinetics and biodistribution of drugs, thus improving the efficacy and security of the therapy [135]. s-PLA can be synthesized using ROP of the lactide with multifunctional initiators. The initiator is a critical factor affecting the properties of s-PLA. A wide range of initiating alcohols has been used, such as glycerol and pentaerythritol, to synthesize three- and four-arm s-PLA respectively, di(trimethylolpropane), glucose and xylitol to synthesize five-arm s-PLA [132,133] and Iron (III) tris(dibenzoylmethane) for six-arm s-PLA synthesis [135]. Star-shaped polyesters were synthesized by reacting l-lactide with glycerol, as an initiator, in the presence of stannous octoate or tetraphenyltin as a catalyst. Three different sizes of three-arm s-PLLA microspheres were synthesized, and the degradation rate was substantially promoted by high glycerol content [136,137]. Erythritol was used as the initiator to produce s-PLLA synthesis microspheres loaded with rifampicin (RIF) as the drug model. Different monomer/initiator molar ratios were used, affecting the morphology of microspheres. The increase of monomers led to a perfectly spherical shape and smooth surface microspheres. Furthermore, the increased amount of monomer results in the preparation of microspheres with a bigger diameter and broader size distribution, sustaining the release of the drug for a considerable period of time (70–80% within 180 h) [129]. S-PDLLAs were also synthesized from pentaerythritol (tetrafunctional: 4-armed polymers) and dipentaerythritol (hexafunctional: 6-armed polymers) as polyol initiators. It was shown that s-PDLLAs degrade via a surface mechanism, with slower kinetics than the bulk-degrading linear ones. s-PDLLAs and linear nanoparticles showed equally high loading efficiency and bioavailability of a model drug, atorvastatin [138]. Glucose was also used as an initiator to formulate s-PLGA microparticles delivering octreotide acetate for up to 1 month. This is the only product of the s-PLA family that has reached the stage of commercial exploitation, known as Sandostatin LAR^®^ Depot [139]. Additionally, s-PLLA was synthesized via ring-opening polymerization of l-lactide, using xylitol, a natural compound, as an initiator to produce microspheres with average diameters between 7 and 15 µm, which could be controlled by varying the s-PLLA’s concentration or Mw. Bovine serum albumin loaded in high molecular weight s-PLLA microspheres exhibited an encapsulation efficiency of 10–42%. Drug-loaded microspheres exhibited low burst release and slow-release rates of bovine serum albumin [140]. It was also proved that the degradation rate of s-PLLA increased with the increase of the xylitol molar fraction [141]. Stereocomplex star-shaped microparticles based on PLA (sc-star-PLA) have also been synthesized between enantiomeric poly(l-lactide) (PLLA) and poly(d-lactide) (PDLA) for targeting drug delivery applications. Interactions between l-lactyl and d-lactyl give better mechanical performance, as well as thermal and hydrolysis resistance, a larger amount of drug adsorption, and slower drug release [142,143,144]. Six arm sc-star-PLA microspheres were synthesized by controlled ring-opening polymerization of l- and d-lactide using dipentaerythritol as the initiator. Carboxylic end-groups were introduced onto the sc-star-PLA, using succinic anhydride, which reacted with OH end-groups. Different sizes of microspheres (1, 2, and 4 mm) were produced by varying the PLA concentration, the increase of which led to particles with increased diameter. sc-star-PLA-OH exhibited porous structures with a worm-like morphology, whereas the star-PLA-COOH formed large spherical objects. Stereocomplexation of star-PLA demonstrated a remarkably high thermal stability, increasing the melting temperature by ~50–60 °C [145]. The higher thermal stability of sc-star-PLAs than star-PLAs was also confirmed by Satoh and co-workers. They reported an innovative method to synthesize sc-star-PLAs by click coupling of azido-functionalized poly-d-lactic acids (PDLAs) and the ethynyl-functionalized PLLAs possessing 4-, 5-, and 6-arms. However, it was also proved that an increase in the number of arms caused a decrease in the Tm and crystallinity of stereocomplexes [146].

s-PLA has been combined with several polymers to tune some of its properties, such as hydrophilicity, drug encapsulation, biodegradability, etc. Star-branched block copolymers of l-PLA and poly(ethylene oxide) PEO were synthesized using ring-opening polymerization with different LA/EO ratios, which were then utilized to prepare micellar aggregates as drug delivery carriers for 5-FU and paclitaxel. The results showed that the degradation of the micellar form of the star-shaped copolymer was much more rapid than the linear diblock copolymer due to the large hydrodynamic volume of PEO. Moreover, it was found that the increase in the length (and relative amount) of the PLA block slowed down the overall 5-FU and paclitaxel release rate [147]. Furthermore, thermoplastic biodegradable hydrogels based on star-shaped PEO-PLA block copolymers with different numbers of arms were synthesized. Albumin was loaded in star shaped microspheres and showed no significant difference between star-polymers for the first 25 days. However, in the latter phase, 8-arm PEO–PLA showed an increase in Albumin release rate, while 2- and 3-arm PEO–PLA showed a decline. Four-arm PEO–PLA showed an in-between release pattern, demonstrating almost zero-order release kinetics [148].

PEG was also used as a hydrophilic component for the preparation of amphiphilic star-shaped block copolymer micelles. Star-shaped PLLA-PEG micelles were synthesized by three stages of chemical reaction. Ibuprofen, as a hydrophobic model drug, was encapsulated into the s-PLLA-PEG micelles, presenting average diameters of micelles between 105 and 121 nm. The drug loading content and encapsulation efficiency were improved by increasing the Mw of PEG, while the drug release behavior could be controlled with different PEG molecular weights [94,149].

A new type of four-armed star-shaped porphyrin-cored poly(lactide)-b-d-α-tocopheryl polyethylene glycol 1000 succinate amphiphilic copolymer (TAPP-PLA-b-TPGS) was synthesized through an arm-first approach for use in drug delivery. Docetaxel loaded in TAPP-PLA-b-TPGS presented excellent pH-dependent drug-release behavior. They were also found to generate singlet oxygen species and exhibit significant phototoxicity in HeLa cervical cancer cells compared to the commercial drug, Taxotere^®^, after irradiation with light of 660 nm wavelength [150]. Star-shaped cholic acid-core polylactide-d-α-tocopheryl polyethylene glycol 1000 succinate (CA-PLA-TPGS) block copolymer was developed for paclitaxel delivery for breast cancer treatment, which demonstrated superior in vitro and in vivo performance with higher antitumor efficacy in comparison with paclitaxel-loaded poly(d,l-lactide-co-glycolide) (PLGA) nanoparticles and linear PLA-TPGS nanoparticles. They also exhibited high stability and showed no change in the particle size and surface charge during 90-day storage of the aqueous solution [151]. The star-shaped PLGA–b-cyclodextrin (PLGA–b-CD) copolymer was synthesized by reacting l-lactide, glycolide, and b-cyclodextrin in the presence of stannous octoate as a catalyst. An antitumor antibiotic, adriamycin, was encapsulated within PLGA–b-CD microspheres with a modified double emulsion method. It was found that the decrease in polymer concentration resulted in increasing the particle average size from 135.5 to 325.6 nm. The entrapment efficiency of adriamycin in 220 mm particles was about 65%, exhibiting a high initial burst and a more rapid release rate [135]. Moreover, unimolecular micelles, each of which is formed by a single amphiphilic macromolecule, were developed. Unimolecular micelles do not dissociate upon dilution and are robust to environmental changes in contrast to conventional block copolymer micelles associated with noncovalent interaction. Specifically, star-like block copolymers, PLA-b-PEG sequentially grafted from β-CD via ring-opening polymerization (ROP) and atom transfer radical polymerization (ATRP) reactions, were reported. These star-like amphiphilic polymers can form monodisperse and stable unimolecular micelles in water. The hydrophobic anticancer drug doxorubicin loaded in the PLA shell was efficiently uptaken by tumor cells, demonstrating pH-controlled release behavior [135].

### 4.6. MPs Prepared by PLA/Poly(Alkylene Adipate) Matrices

**Figure 17:**
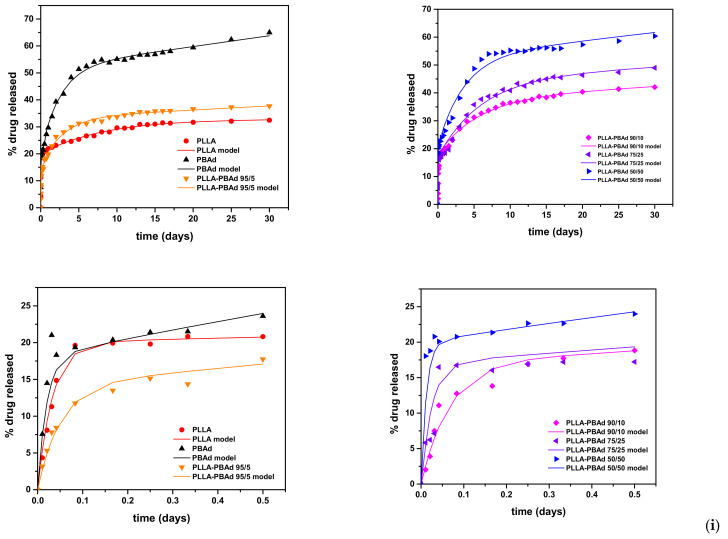

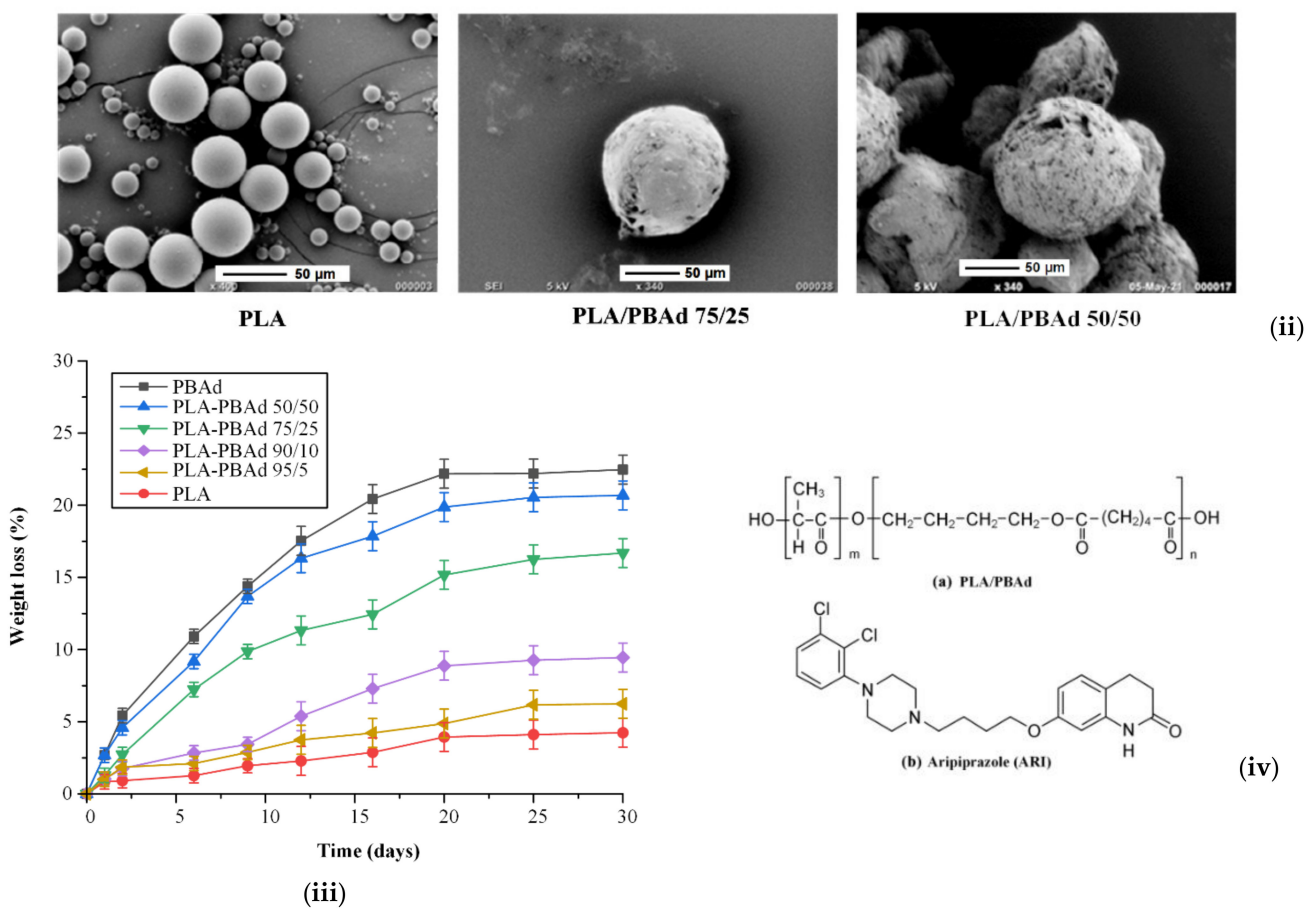
(**i**) Comparison of experimental release data (symbols) to the mechanistic model-based (continuous lines). (**ii**) SEM images of the polyester erosion process after 30 days of dissolution. (**iii**) The enzymatic hydrolysis profile measured as % weight loss vs. time plots for the neat PBAd, the neat PLA, and the various PLA/PBAd block copolymers. (**iv**) Chemical structures of prepared PLA/PBAd copolymer and aripiprazole drug [154].

Lately, several other aliphatic polyesters, such as poly(propylene adipate) [PPAd] and poly(butylene adipate)V [PBAd], have drawn attention in the preparation of depot pharmaceutical formulations, offering an interesting alternative in an effort to improve or replace the characteristics of PLGA. PBAd, as a biodegradable, non-toxic, linear aliphatic polyester, presents quick biodegradation and high thermal stability [152]. These properties constitute PBAd as another eco-friendly candidate for use in biomedical applications, especially for drug delivery. In a recent study, block copolymers of PBAd in combination with PLLA were synthesized in particular with PLLA/PBAd ratios of 95/5, 90/10, 75/25, and 50/50, for the first time by Karava et al. The effect of various PLA to PBAd ratios (95/5, 90/10, 75/25, and 50/50 *w/w*) on the enzymatic hydrolysis of the copolymers showed increasing erosion rates by increasing the PBAd content [153]. The newly synthesized poly(l-lactic acid)-copoly(butylene adipate) (PLA/PBAd) block copolymers were then used as microcarriers for the preparation of aripiprazole (ARI)-loaded long acting injectable (LAI) formulations [154]. Results of the in vitro dissolution studies suggested a highly tunable biphasic extended release for up to 30 days (Figure 17i), which renders the prepared MPs as very promising candidates for new formulations that will probably be able to maintain a continuous therapeutic level for an extended time period and reduced lag-time, as compared to the currently marketed ARI LAI product. SEM images taken after the completion of dissolution proved the crucial role that the amount of PBAd content has to the extent of polyester degradation and consequently the drug release rates. Microspheres with high PBAd content have been extensively eroded (Figure 17ii) compared to PLA microspheres. This is due to the higher hydrolysis rate that PLA/PBAd copolymers have. As can be seen from Figure 17iii, hydrolysis is proportional to the PBAd content, while PLA showed the lowest hydrolysis rate, reaching about 3% within the first six days of testing.

Nanaki et al. focused on the synthesis of a series of novel block copolymers of poly(l-lactide)-block-poly(propylene adipate) (PLLA-b-PPAd) that were further investigated as polymeric matrices for the preparation of naltrexone base (NTX)-loaded microparticle long-acting injectable (LAI) formulations [155].

PPAd is an interesting aliphatic polyester with a low melting point and Tg values, which increases its hydrolysis rate [156]. A naltrexone base is used in the treatment of both drug addiction and alcohol dependence as a specific opioid antagonist. As observed from SEM images, all microparticles had a spherical morphology with smooth surfaces and without any agglomeration formed. However, their morphology after 8 days of dissolution was changed, and from SEM micrographs it was found that their surface became rougher, and the normal spherical shape of the original microspheres was modified. Diffusion was the main mechanism of drug release of these microparticles.

In another study, Nanaki et al. studied the preparation of risperidone-controlled release microspheres as appropriate LAI formulations, this time based on a series of novel biodegradable and biocompatible PLA/PPAd polymer blends prepared by a solvent evaporation method [157]. Risperidone, due to its high hydrophobicity, has a very low dissolution rate, which did not exceed 10% release after six days. However, when risperidone was encapsulated in microspheres, its dissolution was enhanced because it was dispersed in the amorphous phase within the polymer matrices. In vitro drug release studies showed controlled release rates in all PLA/PPAd blend formulations, which is directly dependent on PPAd amount. Dissolution results showed that microspheres consisting of neat PPAd release up to 95% of risperidone within the first 3 days of dissolution, while at the same time only 40% of the API is released by neat PLA microspheres. In microspheres prepared from PLA/PPAd blends the dissolution release rates are between neat polymers (i.e., neat PLA and PPAd). By increasing the PPAd amount in PLA/PPAd blends the API release rate also increased. This was probably due to the low melting point and low glass transition temperatures of PPAd compared to PLA.

### 4.7. PLA/PAsp Copolymer

Poly(aspartic acid) (PAsp) is a hydrophilic fully biodegradable polymer belonging to the family of synthetic polypeptides, insoluble in organic solvents, and can thus be processed only in a hygroscopic powder form or as an aqueous solution. Lately, it has become an attractive candidate for drug carriers. By copolymerization of lactide with aspartic acid, the degradation rate, hydrophilicity, and mechanical and surface properties of PLA can be improved [158]. Tudorachi and his team presented the synthesis of PLA-co-aspartic acid copolymers (PLA-co-Asp), which were tested as biodegradable carriers in drug delivery systems [159]. The PLA-co-Asp copolymers were synthesized by a solution polycondensation procedure, using two different molar ratios PLA/l-aspartic acid (2.33/1, 1/1, 1/2.33). Diclofenac sodium, a non-steroidal anti-inflammatory drug, was subsequently loaded into the PLA-co-Asp copolymers. The PLA-co-Asp/diclofenac sodium systems (DDS_1_, DDS_2_) were prepared by precipitation and solvent evaporation method preparing microparticles, and in vitro drug release experiments were further conducted. It was found that diclofenac can be released much faster from microparticles with lower diameter DDS1 (particles d < 39.4 μm) while a delay was observed from microparticles with high particle size (DDS2) (d < 115.2 μm). Nevertheless, at the end of the dialysis time (356 h), diclofenac sodium released from the DDS1 system was 62.47 wt.% from the whole quantity of drug present in the system, and from DDS2 only 36.09 wt.%.

## 5. Conclusions

Microfabricated systems provide numerous advantages over conventional drug delivery systems. Microcapsules and microspheres are acknowledged as exceptional carrier systems for several drugs and can be tailor-made to adhere to targeted tissue systems. Therefore, microparticle fabrications may find application not only for sustained release, but also for the targeted delivery of drugs to a specific site in the human organism. Although their size can cause some limitations compared to other nanosized vehicles that can aim even at a cellular/subcellular target, microparticles can serve as excellent depot systems for a controlled drug release, and they are able to effectively carry an abundance of (even large) molecules, including polypeptides and proteins.

Polymeric microparticles have been extensively investigated and widely used as DDS. The tailorability of their properties (including crystallinity and degradation rates), the low toxicity, the variety of production techniques, and the ease and low cost of their fabrication render them a powerful DDS design strategy against other materials (e.g., inorganic). The utilization of microparticles based on PLA, PLGA, and related polymers and copolymers in the field of sustained release of therapeutics has lately become a prominent field of research due to their excellent biodegradability and biocompatibility. The available methods to fabricate such systems proved to be very versatile. In this direction, enhanced drug release behaviors, different particle size-structure properties, and loading capacities can be achieved by adjusting some of the experimental variables concerning the preparation process. Despite the significant progress that has been made in the area of microencapsulation, many challenges are still ahead. Significant emphasis should be given to the development of cheaper biopolymers for microencapsulation technology and the development of suitable evaluation techniques, especially for bioadhesive microsystems. Thus, the establishment of harmless and efficient systems will require, in the future, in-depth studies of both the technological and biological features of these systems.

## Figures and Tables

**Table 1:** Overview of advantages and limitations of the SC-CO_2_-based techniques [27].

SCF Technique	Advantages	Limitations
RESS, RESOLV	Single-step particle production. No/low amount of organic solvent required. Final product properties can be tuned by controlling the process parameters. The final product is free of residual solvent.	Solute should be solid or amorphous. The solubility of solute is selective for low molecular weight polymers and small molecules.
SAS, GAS, SEDS	Milder process parameters (temperature and pressure required) compared to RESS. Overcomes the limitation of solute solubility in SC-CO_2_. Encapsulation of labile active substances is possible.	Use of organic solvent. Some biopolymers tend to plasticize in the presence of SC-CO_2_.
PGSS	Organic solvent free process. Homogenous product obtained. Encapsulation of labile active substances is possible.	Particle aggregation may occur during the product formation. Nozzle blockage can occur.
SFEE	Wider range of biopolymers can be processed. Encapsulation of hydrophobic drugs, proteins, and essential oils is possible. Monodisperse particle production.	Multiple steps are required. Organic solvent used.

**Table 2:** Inhalable PLGA microparticles for pulmonary drug delivery systems.

Particles	Drug	Treatment	Reference
PLGA MPs	Rifampicin	Tuberculosis	[77]
PLGA MPs	Levofloxacin	Cystic fibrosis	[78]
Large porous PLGA MPs	Doxorubicin and p53 gene	Lung cancer	[79]
Large porous PLGA MPs	Curcumin	Idiopathic pulmonary fibrosis	[80]
Porous PLGA MPs	Doxorubicin and miR-519c	Lung Cancer	[81]
PEG-modified PLGA	Budesonide	Inflammation in bronchial wall	[82]
PLGA MPS	Salmon Calcitonin	Paget’s disease, Osteoporosis	[76]
Surface-Modified PLGA Microparticles	Gatifloxacin	Tuberculosis	[83]
Large porous PLGA MPs	Sildenafil citrate	Pulmonary arterial hypertension	[84]
PLGA−lipid hybrid MPs	Rifampicin	Antibacterial activity	[85]
PLGA MPs	Rifampicin	Tuberculosis	[77]

**Table 3:** Microparticles from PLA-PEG copolymers for controlled drug delivery.

Particles	Drug	Treatment	Reference
PLLA-PEG MPs	Ibuprofen	Aches and Pains	[94]
PLLA-PEG-Folic acid MPs	Paclitaxel and Doxorubicin	Cancer	[107]
PLA-PEG-PLA MPs	Paclitaxel	Ovarian, breast, colon, head and neck and non-small cell lung cancers	[109]
PLLA-PEG-PLLA	Morphine	Cancer pains	[110]
PLA-PEG-Magnetite MPs	Paclitaxel	Cancer-Hypothermia	[111]
PLA-PEG MPs	Perillyl Alcohol	Glioma	[115]
PEG-PLA MPs	Tat-EGFP	Retinal diseases	[95]
PLA-PEG-PLA MPs	Bone Morphogenetic protein	Bone regeneration	[116]
mPEG-PLA Microspheres	5-Fluorouracil	Cancer	[113]
PLA-PEG-PLA triblock copolymer microspheres	Paclitaxel	Cancer	[112]

**Table 4:** Microparticles from PLGA-PEG copolymers for controlled drug delivery.

Particles	Drug	Treatment	Reference
PLGA-PEG-PLGA -montmorillonite MPs	Aspirin	Inflammations, fever and pain reduction	[96]
PLGA-PEG MPs	Neuregulin (NRG)	Myocardial infarction	[117]
PLGA-PEG-PLGA MPs	Bone morphogenetic protein 2 (BMP-2)	Bone regeneration	[118]
PLGA-PEG	Clonidine	Arterial pressure	[97]
PLGA-PEG	Lysozyme	Tissue engineering	[119]
PLGA-PEG-PLGA MPs	Insulin	Diabetes	[91]

**Table 5:** PLCL microencapsulated formulations for various applications.

Encapsulated Substance	Application	Encapsulation Method	LA/CL Molar Ratio	Advantages	Reference
5-fluorouracil (5-FU)	chemotherapeutic agent	spray drying	PLCL 25:75 PLCL 75:25	Release rates of 5-FU from PLCL somewhat greater than that observed with PLGA/PLA microspheres	[123]
Cyclosporin A (CyA)	prophylaxis and therapy of graft rejection in all types of solid organ and bone marrow transplantation treatment of several autoimmune diseases	oil-in-water (O/W) emulsion solvent evaporation	78.7/21.3 48.1/51.9	Higher blood level of CyA during the initial 2 days, as well as constant levels for several weeks regarding in vivo evaluation	[124]
Nystatin	antifungal drug	oil-in-water (o/w) emulsion solvent evaporation	86 mol% DL-lactide	Stable microspheres and no degradation observed during the period of study of two months	[127]
Ibuprofen	post-operative epidural arthritis dysmenorrhea dental pain	oil-in-water (o/w) solvent evaporation	78.7/21.3	The complete ibuprofen release duration from the microspheres exceeded 1 month	[128]

## Data Availability

Data is contained within the article.
